# Expression of poplar sex-determining gene affects plant drought tolerance and the underlying molecular mechanism

**DOI:** 10.1093/hr/uhaf066

**Published:** 2025-03-05

**Authors:** Jing Lu, Yonghua Yang, Tongming Yin

**Affiliations:** State Key Laboratory for Tree Genetics and Breeding, Co-Innovation Center for Sustainable Forestry in Southern China, Key Laboratory of Tree Genetics and Biotechnology of Educational Department of China, Key Laboratory of Tree Genetics and Breeding of Jiangsu Province, Nanjing Forestry University, No. 159 Longpan Road, Xuanwu District, Nanjing 210037, China; Sainsbury Laboratory, University of Cambridge, 47 Bateman Street, Cambridge CB2 1LR, UK; Institute for Plant Molecular Biology, State Key Laboratory of Pharmaceutical Biotechnology, School of Life Sciences, Nanjing University, 163 Xianlin Avenue, Qixia District, Nanjing 210023, China; State Key Laboratory for Tree Genetics and Breeding, Co-Innovation Center for Sustainable Forestry in Southern China, Key Laboratory of Tree Genetics and Biotechnology of Educational Department of China, Key Laboratory of Tree Genetics and Breeding of Jiangsu Province, Nanjing Forestry University, No. 159 Longpan Road, Xuanwu District, Nanjing 210037, China

## Abstract

It is frequently observed that plant sexes differ in their response to environmental stress. Poplars are dioecious plants, and sex separation of poplars is triggered by the sex-limited expression of the poplar sex-determining gene *FERR*. In this study, we over-expressed *FERR* in a male poplar and knocked it out in a female poplar. The over-expression lines exhibited distinct morphological and physiological changes rendering the transformed plants more tolerant to drought stress. By contrast, no obvious change in drought tolerance was observed in the knockout lines. Transcriptome sequencing and molecular interaction analysis demonstrated that the effect of FERR on drought tolerance was conferred by competitive interaction with protein phosphatase 2C and SNF1-related protein kinase 2 (SnRK2). Under drought stress, an FERR-SnRK2s-ARR5 complex forms and activates the ABA signaling pathway. Our results provide direct evidence that the expression of the poplar sex-determining gene pleiotropically affects plant drought tolerance.

## Introduction

Compared to animals, dioecy is relatively rare in plants and varies greatly in different phyla of *Plantae*. In bryophytes, about 40% of the *Anthocerotae*, 57% of the *Marchantia polymorpha*, and 68% of mosses are dioecious [1] and in gymnosperms, almost 65% of contemporary species are dioecious [2]. Dioecy is recognized in a much lower proportion of angiosperms—only about 6% of the flowering plants are dioecious [3]. It was reported that the occurrence of dioecy correlates with climate, with a much higher frequency in tropical climates [2, 4, 5], which may suggest a sensitivity of dioecious species to environmental changes. It is known that sexes differ in their response to environmental stresses, including drought [6], salinity [7], temperature [8], UV-B radiation [9], nutrient deficiency [10], heavy metals [11], and insect herbivory stress [12]. For example, in *Salix myrtillacea* and *S. paraplesia*, the female trees exhibited higher drought tolerance than the male ones [6, 13]. By contrast, salt stress led to more severe growth limitation in the female *Populus cathayana* than in the males [7]. Under Cd stress, *P. cathayana* females suffered more severe ultra-structural damage than the males [11].

Although dioecious plants were widely observed to show sexual dimorphism in response to different biotic or abiotic stresses, we lack direct mechanistic evidence of this phenomenon. The different sexual responses to environmental stress might be due to sex-linked genes residing in a genomic region with suppressed recombination or could arise from pleiotropic effects of genes affecting primary sexual characteristics [14]. In the recent decade, sex determination genes were identified in several dioecious plant species, such as persimmon [15], garden asparagus [16], kiwifruit [17, 18], and poplar [19, 20], which provided us with the unique opportunity to explore the genetic mechanism underlying the sex-specific responses to environmental stress.

**Figure 1:**
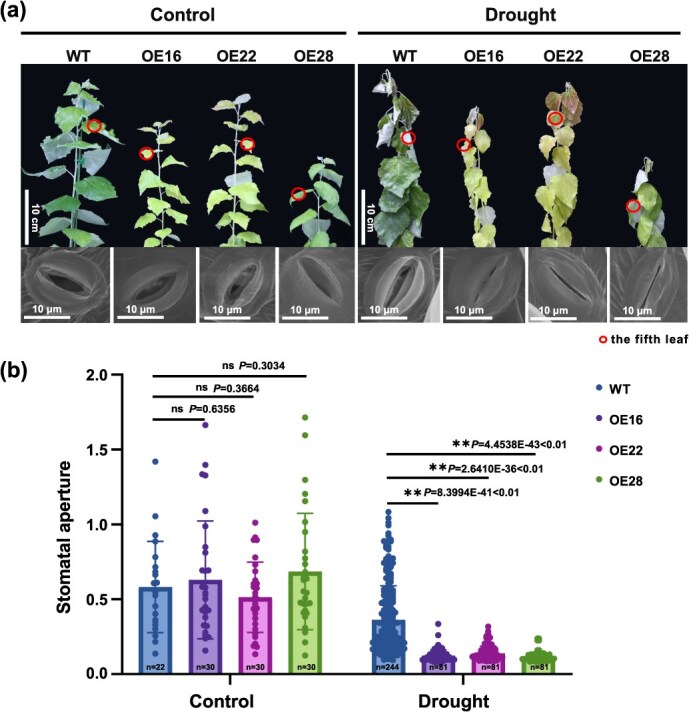
Overexpression of *FERR* reduces water loss by regulating stomatal closure and enhances drought resistance. **(a)** Comparison of drought resistance and stomatal movement between the OE and the WT of ‘84 K’ under well-watered and drought stress conditions. **(b)** Measurement of stomatal aperture with or without drought treatment. Error bars indicate the standard deviations (SDs). Asterisks indicate significant differences from the WT (^*^P < 0.05; ^**^P < 0.01; two-sided Student’s *t*-test), while ns indicates no significant difference (*P* > 0.05). WT: wild type; OE: over-expression line

*P. deltoides* is a dioecious woody species, bearing male or female flowers on alternate sexes of trees. Sex determination of *P. deltoides* occurs through the XY system [19]. In our previous study, we identified the X and Y haplotype blocks in the sex-linked region (SLR). SLR harbors a Y-specific DNA segment, in which the nonprotein coding gene *FERR-R* generates siRNAs to block the expression of the poplar femaleness-promoting gene, *FERR* (homolog to the *Arabidopsis ARR17* gene), which resides in the pseudo-autosomal region of the sex chromosomes [19]. Contrary to the male, the female *P. deltoides* lacks the Y-specific *FERR-R* gene and undisturbed *FERR* expression leads the tree to develop into a female. Knocking out the poplar femaleness-promoting gene in female *P. tremula* results in complete sex change [20]. Therefore, sex-limited expression of *FERR* triggers the differentiation of the male and female polar flowers. In SLR, both the X and Y chromatids possess the genes for T-complex protein 1 subunit gamma, the chloride channel protein L-c, and DNA-methyltransferase 1. Expression analysis showed that the three sex-linked genes are expressed at the same level in the alternate sexes, suggesting that they are less likely to cause the sex-specific response to environmental stress. The aforementioned results made poplar a desirable system to investigate whether the poplar sex-determining gene triggers a pleiotropic effect in the response to environmental stress.

In this paper, we test whether the expression of the poplar sex-determining gene has a pleiotropic effect on plant endurance to environmental stress, specifically drought stress. We over-expressed the poplar sex-determining gene in a male poplar, and knocked it out in a female poplar and examined the responses of both to drought stress. Our results showed that expression of the poplar sex-determining gene can indeed cause pleiotropic effects in the response to drought stress.

**Table 1:** Effect of *FERR* on root biomass in the OE.

**Root biomass**	OE					WT **plants**					***t*-test**
	***n***	**Mean (g)**	**SD.**	**Range**	**CV**	***n***	**Mean (g)**	**SD**	**Range**	**CV**	***P***
FW	15	2.19	0.33	1.74–2.85	0.15	15	1.85	0.15	1.62–2.12	0.08	0.0006
DW	15	0.63	0.09	0.50–0.81	0.15	15	0.47	0.04	0.41–0.55	0.09	0.0000

**Figure 2:**
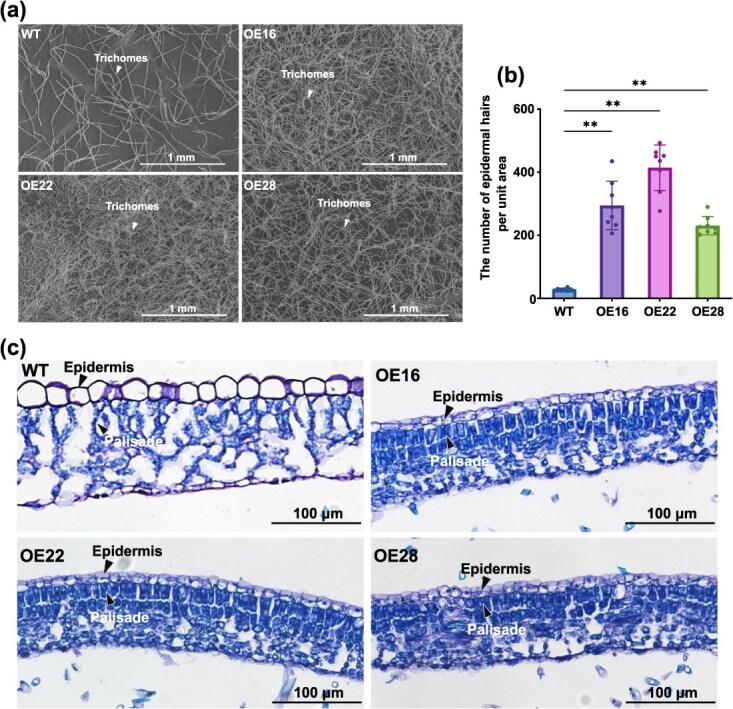
Comparison of trichome density and structure of palisade and epidermis of leaves between the OE and the WT ‘84 K’. **(a)** Scanning electron micrograph of leaf trichome density. **(b)** The number of epidermal hairs per unit area (0.65 × 1.30 mm). Error bars indicate the SDs, with a sample size of 8 per genotype. Asterisks indicate significant differences from the WT (^*^P < 0.05; ^**^P < 0.01, one-way ANOVA with Dunnett’s test). **(c)** Changes in the structure of leaf palisade and epidermis. WT: wild type; OE: over-expression line

## Results

### The pleiotropic effects of the poplar sex-determining gene on drought tolerance

Whether the poplar sex-determining gene affected response to environmental stress was explored by over-expressing the *FERR* gene in a male poplar and by knocking it out in a female poplar. In this study, a total of 139 OE and 91 knockout lines (in 14 editing types, Fig. S2b) were derived. The drought tolerance experiment showed that the OE lines exhibited less leaf curling and wilting than the wild-type (WT) plants under drought stress (the upper panel of Fig. 1a), indicating over-expression of *FERR* could improve plant drought tolerance. By contrast, the knockout lines wilted as the WT plants did when treated by withholding of water (Fig. S3a), suggesting knockout of *FERR* did not affect plant drought tolerance.

Subsequently, we examined the morphological and physiological changes relevant to plant drought tolerance for the OE lines. It was observed that the stomatal aperture was significantly affected by drought stress (the lower panel of Fig. 1a). When well watered, there was no significant difference in the stomatal aperture between the WT plants and OE lines. By contrast, the stomatal aperture exhibited a much sharper decrease in OE (decreased from 0.61 to 0.13) than in WT (decreased from 0.58 to 0.37) (Fig. 1b) under drought stress. Stomatal opening or closure under drought stress greatly affects plant survival [21]. Over-expression of *FERR* led to more stomatal closure in response to drought stress, which would improve plant drought tolerance. It was reported that less reduction in total root biomass in response to drought is indicative of improved drought tolerance [22]. In this study, we measured root biomass for 15 OE lines and 15 WT plants. The statistics revealed that over-expression of *FERR* led to a significant increase in root biomass (Table 1). In contrast, root biomass showed no significant change in the knockout lines (Table S2). Besides, we also observed that the leaf surface of OE was characterized by a dramatically increased density of leaf trichomes (Fig. 2a and b) and smaller and more tightly arranged epidermis and palisade cells than that of WT (Fig. 2c). Conversely, the leaf epidermal and palisade cells in DR exhibit sparse arrangement as in the WT (Fig. S3b). All the above morphological changes can improve plant drought tolerance.

Apart from the morphological changes, we also measured the physiological and biochemical parameters relevant to drought tolerance for the OE lines. Relative water content (RWC) is an important physiological parameter relevant to plant drought tolerance [23]. When well watered, RWC showed no significant difference (*P* > 0.05) between the OE and WT. By contrast, RWC was maintained at a significantly higher level (with a significant *P* value of 3.33E-08, Table 2) in OE (59.44%) than in WT (21.87%) under drought stress. However, no significant changes were observed in the knockout lines (Table S3). For the biochemical parameters, we measured the superoxide dismutase (SOD) and peroxide dismutase (POD) activity, the proline content, and the reactive oxygen species (ROS) accumulation. When plants were well watered, almost all the measured biochemical parameters differed insignificantly (*P* > 0.05) between OE and WT (Table 3, except for POD activity differed significantly at *P* < 0.05) but under drought stress, all the examined biochemical parameters differed significantly between OE and WT (*P* < 0.01), with a significant increase in SOD and POD activities and a significant decrease in ROS accumulation in the OE lines (Table 3). These trends typically suggest that the OE lines have enhanced drought resistance.

**Table 2:** Effect of *FERR* on RWC (%) in leaves of the OE.

**Treatment**	OE					WT **plants**					***t*-test**
	***n***	**Mean**	**SD**	**Range**	**CV**	***n***	**Mean**	**SD**	**Range**	**CV**	***P***
Control	27	96.75	0.32	96.24–97.70	0.00	9	90.66	0.62	90.22–91.98	0.00	0.0533
Drought	27	59.44	0.59	58.13–60.18	0.02	9	21.87	5.55	10.21–28.17	0.25	0.0000

**Table 3:** Effect of *FERR* on biochemical and physiological parameters relevant to drought tolerance for the OE under well-watered and drought stress conditions.

**Parameters (well watered)**	OE					WT **plants**					***t*-test**
	***n***	**Mean**	**SD**	**Range**	**CV**	***n***	**Mean**	**SD**	**Range**	**CV**	***P***
SOD (U/g)	9	128.85	6.30	119.32–138.16	0.05	3	124.46	1.68	122.91–126.24	0.01	0.0871
POD (U/g)	9	8545.64	437.89	8115.09–9301.63	0.05	3	8058.83	239.66	7819.20–8298.51	0.03	0.0470
Proline (μg/g)	9	7.98	0.79	6.41–8.87	0.10	3	7.79	1.56	6.55–7.29	0.20	0.8522
ROS (ng/g)	9	534.72	12.70	511.99–571.47	0.02	3	525.85	22.88	505.34–550.53	0.04	0.5872
**Parameters (drought stress)**	OE					WT **plants**					***t*-test**
	***n***	**Mean**	**SD**	**Range**	**CV**	***n***	**Mean**	**SD.**	**Range**	**CV**	***P***
SOD (U/g)	9	187.00	9.70	169.65–198.14	0.05	3	161.81	1.09	160.88–163.01	0.01	0.0000
POD (U/g)	9	20643.68	1071.86	19094.83–22560.70	0.05	3	18234.72	46.51	18191.78–18284.12	0.00	0.0001
Proline (μg/g)	9	17.24	0.94	15.95–18.73	0.05	3	9.59	1.53	8.28–11.27	0.16	0.0070
ROS (ng/g)	9	597.18	51.61	519.25–659.89	0.09	3	705.71	8.44	696.13–712.03	0.01	0.0001

### The affected genes relevant to drought stress in OE

**Figure 3:**
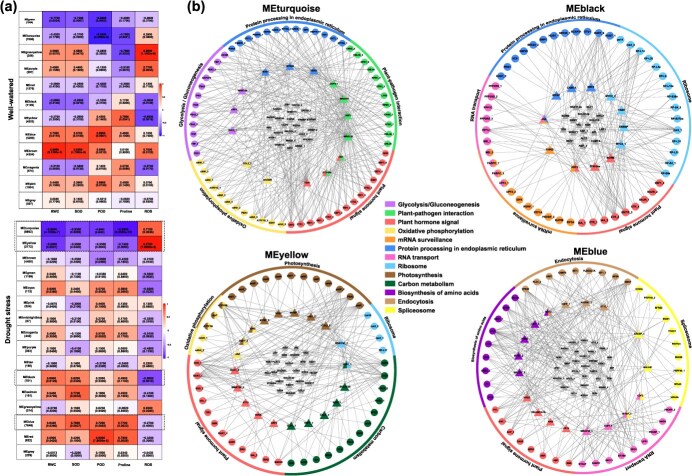
Weighted gene co-expression network analysis of the DEGs relevant to drought tolerance. **(a)** Correlation between co-expression modules and physiological traits. The top and the bottom panels show correlations under conditions of well water and drought stress for OE against the WT plant, respectively. In each panel, the leftmost column is the molecular modules’ identity (the number of genes in each module is listed in the below brackets). The physiological traits are listed at the bottom of each column. The correlation coefficient (upper digital) and the *P* value (lower digital) between modules and physiological traits are listed in the corresponding square. The molecular modules positively or negatively correlated with the measured physiological parameters are labeled out with dashed rectangles (setting significant *P* value <0.05 for at least four of the measured parameters). **(b)** Co-expression network of transcription factors (TFs) and structural genes in molecular modules significantly correlated with drought tolerance. In each panel, the outer colored cycle represents the different KEGG pathways. The dots in the second layer represent the enriched structural genes, and the triangles in the third layer represent the enriched key TFs involved in the corresponding KEGG pathways. In the core of each module, the triangles represent the other TFs involved in the presented KEGG pathways. In the first three layers of each module, the structural genes and the key TFs involved in the same KEGG pathway are displayed in the same color

The transcriptomic data from OE16, OE22, OE28 (the three lines had the highest *FERR* expression, Fig. S4) and WT plants under well-watered and water-withholding conditions showed high quality (Table S4, Fig. S5a). Differential expression analysis identified 899 DEGs under well-watered conditions and 3971 DEGs under drought conditions between WT and OE lines (Fig. S5b). Of these, 617 genes were upregulated and 282 downregulated under well-watered conditions, while 1259 genes were upregulated and 2712 downregulated under drought conditions (Fig. S5c). To further investigate genes relevant to drought tolerance in the OE lines, we extracted DEGs separately by comparing OE lines to WT under well-watered and water-withholding conditions. Correlation analysis with the physiological and biochemical parameters clustered the extracted DEGs into different molecular modules, with 12 and 16 modules established for well-watered and water-withholding conditions respectively (Fig. 3a). Under the water-withholding condition, higher RWC level, higher SOD and POD activities, and higher proline content, but lower ROS accumulation was expected in OE compared to WT. Weighted gene co-expression network analysis (WGCNA) revealed two negatively regulated modules (MEturquoise and MEyellow) and two positively regulated modules (MEblack and MEblue) that were significantly correlated with the physiological and biochemical parameters. Construction of a co-expression network assigned the DEGs in each of the four modules into different KEGG pathways, and results showed that the plant hormone signaling was the most frequently affected pathway (Fig. 3b).

**Figure 4:**
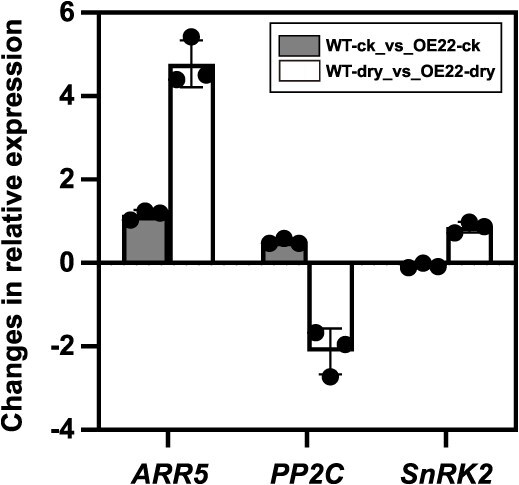
qRT-PCR verification analysis of expression levels of selected DEGs in RNA-seq sequencing data from the WT and OE line of ‘OE22’ under conditions of well water and drought stress. *Ubiquitin* (*UBQ*, [26]) was used as an internal control, and data are presented as FC (*n* = 3 per group)

PlantCARE analysis of the *FERR* promoter sequence revealed the presence of two ABA response elements (ABRE) (Fig. S6), suggesting *FERR* might affect plant drought tolerance through the ABRE-dependent ABA signaling pathway. A detailed examination of the DEGs in the four significantly correlated modules identified 57 up-regulated and 96 down-regulated genes that were known genes relevant to drought tolerance based on functional annotation (Fig. S5d). Among these DEGs, functional annotation discovered 17 up-regulated and 1 down-regulated DEGs that were involved in ABA signaling (Fig. S5d). A key gene of the ABA signaling pathway [24], *PP2C* (*Pag.A01G001094*), was significantly down-regulated in our analysis. *PP2C* acts upstream of *SnRK2s* in the ABA signaling pathway. Because it is known that *PP2C* can increase the expression of *SnRK2* [25], we checked the expression of *SnRK2s* in our data set. The data showed that two homologous copies of *SnRK2s* in poplar (*Pag.A04G002172* and *Pag.B04G002139*) showed increased expression (Table S5). The elevated expression of *Pag.A04G002172* and *Pag.B04G002139* and down-regulation of *Pag.A01G001094* were further confirmed by qRT-PCR (Fig. 4). *ARR5* is another key gene regulating ABA signaling [24]. Our data revealed that the expression of the *ARR5* homolog *Pag.A01G000486* was significantly up-regulated in OE. To illustrate how FERR affects ABA signaling, we subsequently investigated the protein interactions between FERR and the aforementioned key molecules in the ABA signaling pathway.

### Formation of FERR-SnRK2s-ARR5 complex

**Figure 5:**
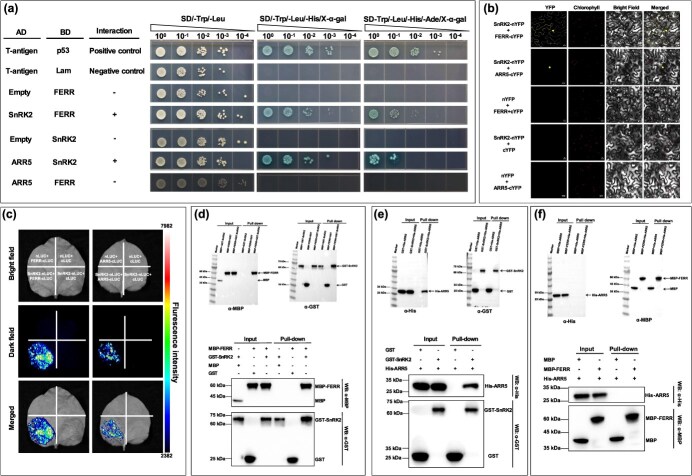
Confirmation of the FERR-Pag.A04G002172 (SnRK2s)-Pag.A01G000486 (ARR5) complex through *in vitro* and *in vivo* interaction assays. **(a)** Y2H assay verified interaction between FERR and Pag.A04G002172, and interaction between SnRK2 and ARR5. AD: prey; BD: bait; Positive control: co-transformation of T-antigen/p53; Negative control: co-transformation of T-antigen/Lam; ‘+’: interaction; ‘-’: no interaction. **(b)** BiFC assay showed interaction between FERR and Pag.A04G002172, and interaction between Pag.A04G002172 and Pag.A01G000486 in the epidermal cells of *Nicotiana benthamiana* leaves. YFP: yellow fluorescent protein; empty nYFP and cYFP vectors used as negative controls. Images are selected as representative. **(c)** Split-LUC transient expression assay assessed the physical interaction between FERR and Pag.A04G002172, and the physical interaction between Pag.A04G002172 and Pag.A01G000486 in the epidermal cells of *N*. *benthamiana* leaves. Empty nLUC and cLUC vectors were used as negative controls. **(d–f)** Pull-down assay tested interaction between FERR and Pag.A04G002172, and interaction between Pag.A04G002172 and Pag.A01G000486, but no interaction between Pag.A01G000486 and FERR. MBP: maltose-binding protein; GST: glutathione S-transferase; His: histidine; WB: western blot. The fusion proteins MBP-FERR, GST-SnRK2, and His-ARR5 were purified and detected by Western blotting. MBP and GST proteins were used as controls

Homology modeling of protein structures of FERR, Pag.A04G002172 (SnRK2s), and Pag.A01G000486 (ARR5) revealed that they lacked transmembrane domains and signal peptides. Experimental tests demonstrated that these genes did not exhibit auto-activation activity. The yeast two-hybrid (Y2H) assay detected that Pag.A04G002172 (SnRK2s) interacted with FERR, and the interaction was confirmed by the yeast-spotting assay (Fig. 5a). The yeast spot assay also revealed a physical interaction between Pag.A04G002172 (SnRK2s) and Pag.A01G000486 (ARR5) (Fig. 5a). In the yeast spot and pull-down assays, we detected no physical interaction between FERR and Pag.A01G000486 (ARR5) (Fig. 5a and f). To confirm the protein interactions, we further conducted *in vivo* biomolecular fluorescence complementation (BiFC) and split-LUC assays, and *in vitro* pull-down assay (Fig. 5b-e). In previous studies, *FERR* and *SnRK2* were localized in both the nucleus and cytoplasm [27, 28], while *ARR5* was specifically localized in the nucleus [29]. In the BiFC assay, when Pag.A04G002172-nYFP and FERR-cYFP were co-expressed, YFP molecules were observed to predominantly reconstitute in the nucleus and cytoplasm (Fig. 5b). Similarly, when Pag.A04G002172-nYFP and Pag.A01G000486-cYFP were co-expressed, functional YFP molecules were merely presented in the nucleus (Fig. 5b). In the split-luciferase (split-LUC) assay, the fusion of FERR-cLUC with Pag.A04G002172-nLUC, as well as the fusion of Pag.A01G000486-cLUC with Pag.A04G002172-nLUC, showed significant LUC signals in *Nicotiana benthamiana* (Fig. 5c). On the contrary, the control tests yielded no detectable LUC signals (Fig. 5c). In the pull-down assay, the MBP-FERR fusion protein in the input and experimental group was detected through the α-MBP antibody, while the GST-Pag.A04G002172 fusion protein was detected through the α-GST antibody (Fig. 5d). Western blot (WB) analysis revealed the luminescence signal only in the group where MBP-FERR and GST-Pag.A04G002172 were incubated together, while no band was detected in the groups incubated with GST-Pag.A04G002172 and MBP, as well as MBP-FERR and GST (Fig. 5d), indicating that GST-Pag.A04G002172 was able to pull down MBP-FERR. Similarly, it was observed that His-Pag.A01G000486 was able to pull down GST-Pag.A04G002172 (Fig. 5e). Collectively, both the *in vivo* and *in vitro* experiments confirmed the protein interaction between FERR and Pag.A04G002172 (SnRK2s), and the protein interaction between Pag.A04G002172 (SnRK2s) and Pag.A01G000486 (ARR5). This indicates that FERR can affect the ABA signaling pathway through the formation of an FERR-SnRK2s (Pag.A04G002172)-ARR5 (Pag.A01G000486) complex, in which SnRK2s acts as the mediating molecule.

### P‌P2C and SnRK2 competitively interact with FERR

**Figure 6:**
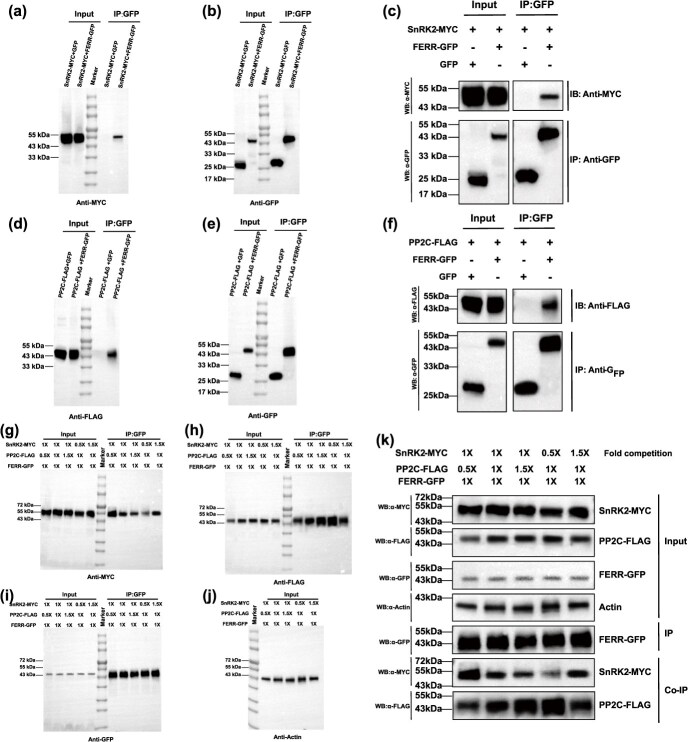
Confirmation of competitive interactions between PP2C and SnRK2 with FERR using co-immunoprecipitation (Co-IP) and competitive binding assays. **(a-c)** Confirmation of the interaction between SnRK2 and FERR using the Co-IP assay. **(d-f)** Confirmation of the interaction between PP2C and FERR using the Co-IP assay. **(g-k)** Confirmation that PP2C competes with SnRK2 for interaction with FERR using a competitive binding assay. Protein extracts (input) were immunoprecipitated with anti-GFP or anti-FLAG beads and resolved by SDS-PAGE. Protein–protein interactions were immunodetected using anti-GFP, anti-MYC, and anti-FLAG antibodies. The concentration of FERR-GFP protein was maintained at 1-fold, while the protein concentrations of PP2C-FLAG and SnRK2-MYC were varied at 0.5-fold, 1-fold, and 1.5-fold to produce five combinations. *Anti*-*actin* was used as a loading control

Under drought stress, *Pag.A01G001094* (*PP2C*) and *Pag.A04G002172* (*SnRK2*) exhibited antagonistic expression, suggesting proteins coded by the two genes might competitively interact with FERR. Physical interaction between SnRK2 and PP2C is conserved [30]. To understand if PP2C and SnRK2 competitively interact with FERR, we used separate co-immunoprecipitation (Co-IP) assays. To confirm the interaction between FERR and Pag.A04G002172 (SnRK2), the experiments were divided into two groups: the input group and the IP group (Fig. 6a-c). The input group consisted of SnRK2-MYC + GFP (control) and SnRK2-MYC + FERR-GFP, while the IP group included SnRK2-MYC + GFP (control) and SnRK2-MYC + FERR-GFP. Immunoblotting with the MYC antibody revealed the desired bands appeared in the input group (Fig. 6a and c). In the IP group, the desired band was also detected for SnRK2-MYC + FERR-GFP, while no band was observed in the control, providing compelling evidence for the interaction between FERR and SnRK2 (Fig. 6a and c). Subsequently, the successful detection of the desired bands using the GFP antibody confirmed the success of the IP experiment (Fig. 6b and c). Similarly, the interaction between FERR and PP2C was detected using the same method described above (Fig. 6d-f). These results indicated the formation of the FERR-PP2C-SnRK2 complex.

The formation of the FERR-PP2C-SnRK2 and the FERR-SnRK2s-ARR5 complex suggested that PP2C or SnRK2 might competitively interact with FERR. To test this hypothesis, we designed five different combinations of experiments (Fig. 6g-k). In the input groups, the desired bands were detected using MYC, FLAG, GFP, and actin antibodies. In the Co-IP groups, when FERR and PP2C were quantified while SnRK2 was variable, the signal of SnRK2 gradually weakened with an increase in PP2C concentration. Similarly, when FERR and PP2C were quantified, and SnRK2 was variable, the signal of PP2C gradually diminished with an increasing concentration of SnRK2 (Figs 6g-k and  S7). Taken together, the Co-IP assays revealed that PP2C and SnRK2 compete to interact with FERR *in vivo*, possibly on overlapping interaction sites on FERR.

## Discussion

For dioecious plants, the separation of sexes is triggered by the sex-determining gene, which leads to the abortion of the alternate sexual organs at different developmental stages of flowers [31]. Transgenic studies showed that the plant sex-determining gene directly affected the characteristics of sexual organs [15, 16, 20]. However, we did on have any direct evidence whether the expression of the plant sex-determining gene affects non-reproductive traits. To address this interesting question, we examined the effects of the plant sex-determining gene in poplar OE and knockout lines. *FERR* in *Populus deltoides* functions as the femaleness promoting gene, and its sexually limited expression determines the formation of male or female floral primordial [19]. This study shows that overexpression of *FERR* in male poplar significantly enhanced drought tolerance, and the elevated drought tolerance was related to a series of morphological, physiological, and biochemical changes, assigning a previously unknown role to *FERR* that goes beyond sex determination. It was observed that OE lines were characterized with more stomatal closure than the WT plants under drought stress. It is known that stomatal opening facilitates leaf photosynthesis, plant growth, and water utilization, whereas stomata closure determines plant survival under drought stress [21]. The leaf surface of the OE lines was characterized by a higher density of leaf trichomes, and smaller and more tightly arranged epidermis and palisade cells than the WT plants. This observation is consistent with the scenario that plants growing in arid environments commonly display tight epidermis and palisade cell arrangements, and a high density of epidermis appendages like leaf trichomes [32, 33]. Root biomass is another indicator of plant drought tolerance [22]. The over-expression of *FERR* led to a significant increase both in fresh and dry root biomass. Besides the morphological changes relevant to plant drought tolerance, the higher drought tolerance of OE was also achieved through improvement in water-keeping ability, increases of antioxidant enzyme activities (SOD and POD), promotion of proline accumulation, and enhancement of ROS scavenging under drought stress. Taking together, multiple lines of evidence showed that *FERR* expression rendered plants more tolerant to drought stress, which might potentially link to higher water requirements in female reproduction [34].

It is well-established that the ABA signaling pathway greatly affects plant drought tolerance [35, 36]. ABA accumulation in response to drought stress leads to stomatal closure, adaptive physiological responses, and changes in gene expression [37, 38]. The *FERR* promoter sequence is characterized by two ABA response elements, and thus may affect the plant ABA signaling pathway. Furthermore, analysis of the transcriptomic data of OE under drought stress revealed distinct clues that *FERR* expression might invoke a pleiotropic effect on ABA signaling. *FERR* is a type-A *RR* gene that affects plant development, such as flower differentiation, through cytokinin signaling [19]. A previous study demonstrated crosstalk between ABA and cytokinin signaling in plant development and drought resistance [39]. Drought stress leads to a reduction in cytokinin synthesis and activation of ABA biosynthesis [40]. We observed that *FERR* over-expression resulted in down-regulation *Pag.A12G000106* (the type-B *RR*). The type-B *RRs* act as positive regulators of cytokinin signaling, while inhibiting the negative regulation imposed by type-A *RRs* [41]. Indeed, with the down-regulation of *Pag.A12G000106*, increased expression of a type-A *RR* gene, *Pag.A01G000486* (*ARR5*) was detected. According to Huang et al.’s study, ARR5 positively regulates ABA signaling through an SnRK2-dependent mechanism [39], and SnRK2 kinases in the ABA signaling pathway interacted with the negative regulators of cytokinin signaling. We propose that FERR might be the mediator molecule between the two signaling pathways.

**Figure 7:**
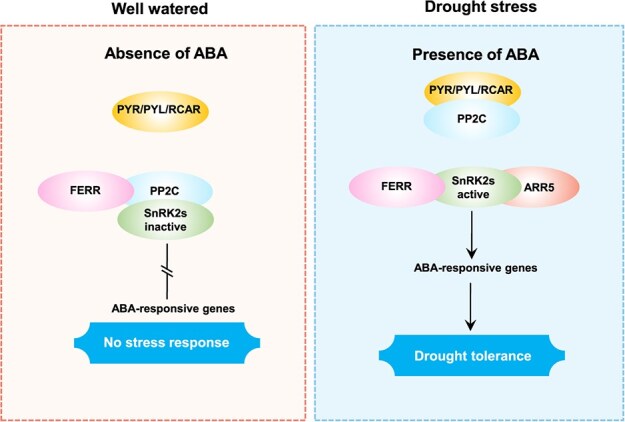
Working model for ABA signaling mediated by competitive interactions between FERR-PP2C-SnRK2s or FERR-SnRK2s-ARR5 complex under of well-watered and drought stress conditions

In our current understanding of ABA signaling, in the absence of ABA, PP2C binds SnRK2s and prevents its activation. Inactive SnRK2 cannot phosphorylate downstream substrates, and thus impedes signal transduction. In the presence of ABA, the ABA receptors (PYR/PYL/RCAR) bind ABA, facilitating their interaction with PP2C, which interferes with PP2C binding to SnRK2s and results in SnRK2s activation. The activated SnRK2s, in turn, regulate downstream gene expression [24]. In this study, multiple lines of evidence confirmed that FERR can bind PP2C and SnRK2s, and that the two proteins competitively interacted with FERR. We propose a working model for ABA signaling mediated by competitive interactions between FERR and PP2C or between FERR and SnRK2s (Fig. 7). In the absence of ABA, PP2C preemptively binds with SnRK2s, and thus blocks the binding between FERR and SnRK2s, resulting in the formation of FERR-PP2C-SnRK2s. In this case, SnRK2s is inactivated, unable to interact with ARR5, and expression of the downstream genes is not affected. Under drought stress, PP2C is bound by the ABA receptors (PYR/PYL/RCAR), which prevents PP2C from binding to SnRK2s, and in consequence facilitates the interaction between SnRK2s and FERR and the interaction between SnRK2s and ARR5, resulting in the formation of the FERR-SnRK2s-ARR5 complex. In this case, SnRK2s kinase is active. Therefore, *FERR* over-expression can improve plant drought tolerance by positively regulating ABA signaling through the formation of the FERR-SnRK2s-ARR5 complex.

Overall, this study provided direct evidence that, in addition to its role in sex organs development, expression of the poplar sex-determining gene also improves the plant drought tolerance by interfering with the ABA signal transduction. Based on our data, we present a model on how this is achieved involving competitive binding and formation of a multiprotein complex directly rendering the ABA pathway active or inactive.

## Materials and methods

### Plant materials and generation of transgenic plants

In male poplars, the expression level of the sex-determining gene *FERR* increases under moderate drought conditions (40%–50% SRWC, soil RWC) compared with well-watered conditions (70%–75% SRWC). Conversely, the opposite trend was observed in female poplars (Fig. S1a and b). Based on this observation, a male poplar, ‘84 K’ (*Populus alba* × *P*. *tremula* var. *glandulosa*), was used in the over-expression transformation. Whereas a female poplar, ‘Nanlin 895’ (*P*. *deltoides* × *P*. *euramericana*), was employed in the CRISPR/Cas9-mediated knockout. The poplar sex determining gene, *FERR*, was cloned from ‘Nanlin 895’. The over-expression vector (35S::*FERR*-OE, Fig. S2a) was constructed with pCAMBIA2301-35Splus through homologous recombination using the *pEASY*®-Uni Seamless Cloning and Assembly Kit (TransGen Biotech Co. Ltd., Beijing, China). The knockout vector for *FERR* (*ferr*-DR, Fig. S2a) was constructed with a CRISPR/Cas9-mediated knockout vector pKSE401 using the Golden Gate cloning method [42]. CRISPR-P 2.0 (http://cbi.hzau.edu.cn/CRISPR2/help.php) and CRISPR-GE (http://skl.scau.edu.cn/) were utilized to identify the CRISPR/Cas9 target sites of the *FERR* gene. Three sgRNAs were incorporated between the *Bsa*I sites of pKSE401.

Transgenic poplars were generated using the *Agrobacterium*-mediated method. Briefly, leaf disks of 84 K were transformed with *Agrobacterium tumefaciens* strain EHA105 carrying the over-expression vector 35S::*FERR*-OE, and leaf disks of ‘Nanlin 895’ were transformed with the same *Agrobacterium* strain carrying the CRISPR/Cas9-mediated knockout vector *ferr*-DR. The screening media was 1/2 Murashige and Skoog (MS) containing 15 mg/L kanamycin (kan) for over-expression transformation, and 50 mg/L kan for CRISPR/Cas9-mediated knockout. The infected explants were rinsed several times with sterilized water after three days in the dark, then were incubated in a growth chamber under 22°C, 16-h-light/8-h-dark, 70 μmol m^−2^ s^−1^ luminous source, and 45% humidity. When shoots elongated to about 1 cm, the plants were transplanted to the rooting medium. After reaching 6 cm in height, the plants were transplanted into pots (in January 2022) filled with a mixture of nutrient soil, loess, and organic substance in a ratio of 3:3:1. Subsequently, transgenic plants were cultivated in a growth chamber with a 16-hour light/8-hour dark cycle, luminous source of 200 μmol m^−2^ s^−1^, and humidity of 80%, at 23°C/15°C day/night temperatures.

Over-expression lines (35S::*FERR*-OE) were confirmed with specific primers (Table S1) designed to amplify the kanamycin resistance (kan) gene in transgenic plants. The amplified PCR products were subjected to Sanger sequencing, and the expression level of *FERR* was quantified using qRT-PCR. Total RNA was extracted from leaves of kanamycin-resistant plants by using the RNAprep Pure Plant Kit (TIANGEN, China) and reversely transcribed into cDNA by using the FastKing gDNA Dispelling RT SuperMix (TIANGEN, China). qRT-PCR was performed by using the PowerUp™ SYBR™ Green Master Mix (Applied Biosystems, USA) on a 7500 Fast Real-Time PCR System (Applied Biosystems, USA). Three lines with the highest *FERR* expression levels were subjected for the subsequent drought-resistant experiments. To identify CRISPR/Cas9-mediated mutants (*ferr*-DR), specific primers (Table S1) were designed to amplify the target editing sites. PCR libraries were sequenced on an Illumina HiSeq 2000 (Illumina, San Diego, CA, USA) by Sangon Biotech Co. Ltd. (Shanghai, China). In-house Perl scripts were used to separate read counts for each sample, and the mutation rate was calculated based on the ratio of the mutation line amplicons against the total sequencing amplicons.

### Drought treatment and drought tolerance assessment

After 120 days of transplanting, seedlings of transgenic lines (27 over-expression ‘84 K’ and 27 knockout ‘Nanlin 895’) and WT (nine ‘84 K’ and nine ‘Nanlin 895’) plants were transplanted into new pots filled with soil that had been dried at 60°C for one week, and grew under condition by withholding water for three consecutive days.

Morphological changes were characterized by leaf structure and root biomass. To observe the changes in leaf palisade and epidermis structures between the OE and WT plants, we collected the fifth leaves from the apical buds on five OE and five WT plants, and leaf structure was compared by observing two areas of the longitudinal sections of each leaf using an Axio Imager 2 microscope (Zeiss, Oberkochen, Germany). With these leaf samples, stomatal movement and leaf trichomes density were determined by observing two areas of the abaxial surface of each leaf using an FEI Quanta 200 scanning electron microscope (Thermos Scientific, Dawson, USA). At least 20 stomata were measured for each genotype and condition. Stomatal apertures were measured from images using the ImageJ/Fiji platform [43] and by following the methods described by Wu et al. [44]. Fresh and dry root biomass was recorded for seedlings of the tested plants using the electronic balance (Sartorius, Germany, sensitivity 0.001 g).

Physiological indices relevant to drought tolerance were assessed by analyzing RWC, SOD, and POD activities, proline content, and ROS levels. RWC was measured by weighing detached leaves for the fresh weight (FW), determining the saturated fresh weight (SW) after water-bathing for six hours, and obtaining constant dry weight (DW) after drying at 80°C for three days. The physiological parameters were measured at Genepioneer Biotech Co., Ltd (Nanjing, China), with three biological repeats and each repeat included three experimental measurements.

### Transcriptome sequencing and analyzing

RNA sequencing (RNA-seq) was performed to analyze the differentially expressed genes (DEGs) relevant to the drought stress. Clonal trees were separately generated with transgenic lines of OE16, OE22, OE28, and the WT ‘84 K’, and RNA-seq was conducted with three trees (biological repeats) for each of them. The fifth leaf from the apical buds on each tree was collected, and subjected to mRNA extraction and cDNA transcription following the description by Hao et al. [45]. Transcriptome sequencing was conducted using the Illumina Novaseq 6000 platform (Illumina, San Diego, CA, USA), with three biological repeats, and each biological repeat included three experimental repeats.

Raw reads were preprocessed using in-house Perl scripts to remove adapters and low-quality sequences. High-quality clean reads were then mapped to the reference genome of ‘84 K’ (https://figshare.com/articles/dataset/84K_genome_zip/12369209) using HISAT2 v2.1.0 [46]. Transcript abundance was quantified using the fragments per kilobase of transcript per million fragments mapped (FPKM) method [47]. DEGs analysis was performed by using the DESeq R Bioconductor package, with a fold change (FC) ≥ 1, and false discovery rate < 0.05 [48]. The heatmap clustering was performed using the pheatmap R package [49]. Gene annotation was performed against databases including NR (http://ncbi.nlm.nih.gov/), Gene Ontology (GO) (http://www.geeontology.org), Kyoto Encyclopedia of Genes and Genomes (KEGG) (http://www.kegg.jp), COG/KOG (https://www.ncbi.nlm.nih.gov/COG/), Uniprot (http://www.uniprot.org/), and Pfam (http://pfam.xfam.org/). GO analysis was performed by using the GOseq R package with a hypergeometric distribution method [50]. The KEGG pathway was predicated using the KOBAS software [51]. To elucidate the gene regulatory network, we integrated the relationship between co-expression modules and physiological indicators by performing a WGCNA with the WGCNA R package v1.72 [52]. The co-expression network was visualized by Cytoscape v3.9.1 [53].

### Y2H assay

The full-length coding sequence of *FERR* from *P. deltoides* was amplified and cloned into the pGBKT7 vector to generate pGBKT7-FERR as bait to screen the interacted proteins from the *P. deltoides* cDNA yeast library by using the Matchmaker™ GAL4 two-hybrid system 3 (Clontech Laboratories Inc., CA, USA). The full-length coding sequence of *SnRK2* and *ARR5* from 84 K were amplified and cloned into vector pGADT7 as prey or pGBKT7 as bait, resulting in recombinant plasmids pGADT7-SnRK2, pGBKT7-SnRK2, and pGADT7-ARR5. Pairwise combinations of pGBKT7-FERR, pGADT7-SnRK2, pGBKT7-SnRK2, and pGADT7-ARR5 plasmids were co-transformed into yeast strain AH109 by using the LiAc/SSDNA/PEG method following the description by Gietz and Schiestl [54]. The transformed yeast cells were then cultured on the synthetic defined medium (SD/−Trp/−Leu). After 72 hours, the yeast cells were spotted onto SD/−Trp/−Leu/-His and SD/−Trp/−Leu/-His/−Ade media with X-α-gal to detect protein interactions. Each culture was diluted in 10^0^, 10^−1^, 10^−2^, 10^−3^, and 10^−4^.

### BiFC assay

The coding regions of *SnRK2* (without stop codons) were inserted into PpSm35s-nYFP to generate SnRK2-nYFP; and the coding regions of *FERR* and *ARR5* were ligated into PpSm35s-cYFP to generate FERR-cYFP and ARR5-cYFP, respectively. These plasmids were transferred into *A. tumefaciens* strain GV3101 and pairwise combinations of vectors were co-agroinfiltrated into the epidermal cells of *Nicotiana benthamiana* leaves. After 48 h, the YFP fluorescence signals were analyzed using an LSM 710 confocal microscope (Carl Zeiss Jena, Oberkochen, Germany).

### Split-LUC assay

The coding regions of *SnRK2* (without stop codons) were fused in-frame with pCambia1300-nLUC, resulting in SnRK2-nLUC. Similarly, the coding regions of *FERR* and *ARR5* were inserted into pCambia1300-cLUC, generating FERR-cLUC and ARR5-cLUC, respectively. The mixture of Agrobacterium suspensions containing pairwise combinations of plasmids was co-agroinfiltrated into the epidermal cells of *N. benthamiana* leaves. After 48 h, the leaves were sprayed with 1 mM luciferin (Promega, Madison, WI, USA), and then incubated for an additional 7 min in the dark. Fluorescence signals were captured and analyzed by the chemiluminescent imaging system (Tanon, Shanghai, China).

### Pull-down assay

The codon-optimized cDNA sequence of *FERR* was inserted into the pMAL-c5x vector with an N-terminus maltose-binding protein (MBP) tag (pMAL-c5x-FERR, MBP-FERR). The coding region of *SnRK2* was inserted into the pGEX-6–1 vector to generate a fusion protein with glutathione S-transferase (GST) tag (pGEX-6p-1-SnRK2, GST-SnRK2). The coding region of *ARR5* was inserted into the pET28a (+) vector to produce a histidine-tagged (His-tagged) fusion protein (pET28a-ARR5, His-ARR5). Each protein was expressed and purified from *Escherichia coli* strain BL21. The recombinant fusion proteins were mixed in equal volumes, incubated, and purified on a GST column. The mixed proteins were washed five times in the washing buffer, eluted, and subjected to Western blot assays with α-MBP or α-GST antibodies (Epitomics, Burlingame, CA, USA).

### Co-IP assay

The coding region of *PP2C* with a 3 × Flag tag was inserted into the pBWA(V)Hs-TMVΩ vector (pBWA(V)Hs-TMVΩ-PP2C-3 × flag, PP2C-FLAG). Similarly, the coding region of *SnRK2* with an MYC tag was inserted into the pBWA(V)Hs-TMVΩ vector (pBWA(V)Hs-TMVΩ-SnRK2–4 × myc, SnRK2-MYC). Additionally, the coding region of *FERR* with a GFP tag was inserted into the pK7WGF2 vector (pK7WGF2-FERR-GFP, FERR-GFP). The empty binary vector pK7WGF2-GFP was used as the negative control. These vectors were transferred into *A. tumefaciens* (GV3101) and then injected into the epidermal cells of *N. benthamiana* leaves. According to Lin and Lai [55], after 48 h, total proteins were extracted and cell lysates were prepared using IP lysis buffer (Thermo, 87 787) supplemented with protease inhibitor cocktails (Yeasen, 36403ES08). Anti-FLAG (MBL, M185-3 L), anti-MYC (Abclonal, AE070), or anti-GFP (Roche, 11 814 460 001) conjugated to protein A/G beads (Yeasen, 36403ES08) for 1 h, washed three times with IP lysis buffer and eluted. The immunoprecipitates were then subjected to SDS–PAGE and immunoblotted with the corresponding antibodies.

### Competitive binding assay

The protocol described by Rani et al. [56] and Ries et al. [57] was followed for the competition co-IP assay. In brief, the concentration of FERR-GFP protein was maintained at 1-fold, while the protein concentrations of PP2C-FLAG and SnRK2-MYC were varied at 0.5-fold, 1-fold, and 1.5-fold to achieve dose-dependent accumulation, resulting in five different combinations. Anti-actin was used as a loading control. Western blotting quantification was measured using ImageJ/Fiji software (National Institutes of Health, Bethesda, NIH) to compare the protein expression levels. The expression levels of the proteins of interest were standardized to the protein expression level of a loading control (e.g. β-actin).

Primers used in these assays are listed in Table S1. All *in vivo* and *in vitro* protein–protein interaction assays were repeated three times.

## Supplementary Material

Web_Material_uhaf066

## Data Availability

The data underlying this article are available in the article and in its online supplementary material.
