# Supplementary material for: Expression of poplar sex-determining gene affects plant drought tolerance and the underlying molecular mechanism
Source: Hortic Res. 2025 Mar 5;12(6):uhaf066. doi: 10.1093/hr/uhaf066 (PMC12038252; doi:10.1093/hr/uhaf066)
Supplement: Web_Material_uhaf066 [file web_material_uhaf066.zip › Figure S2.pdf]

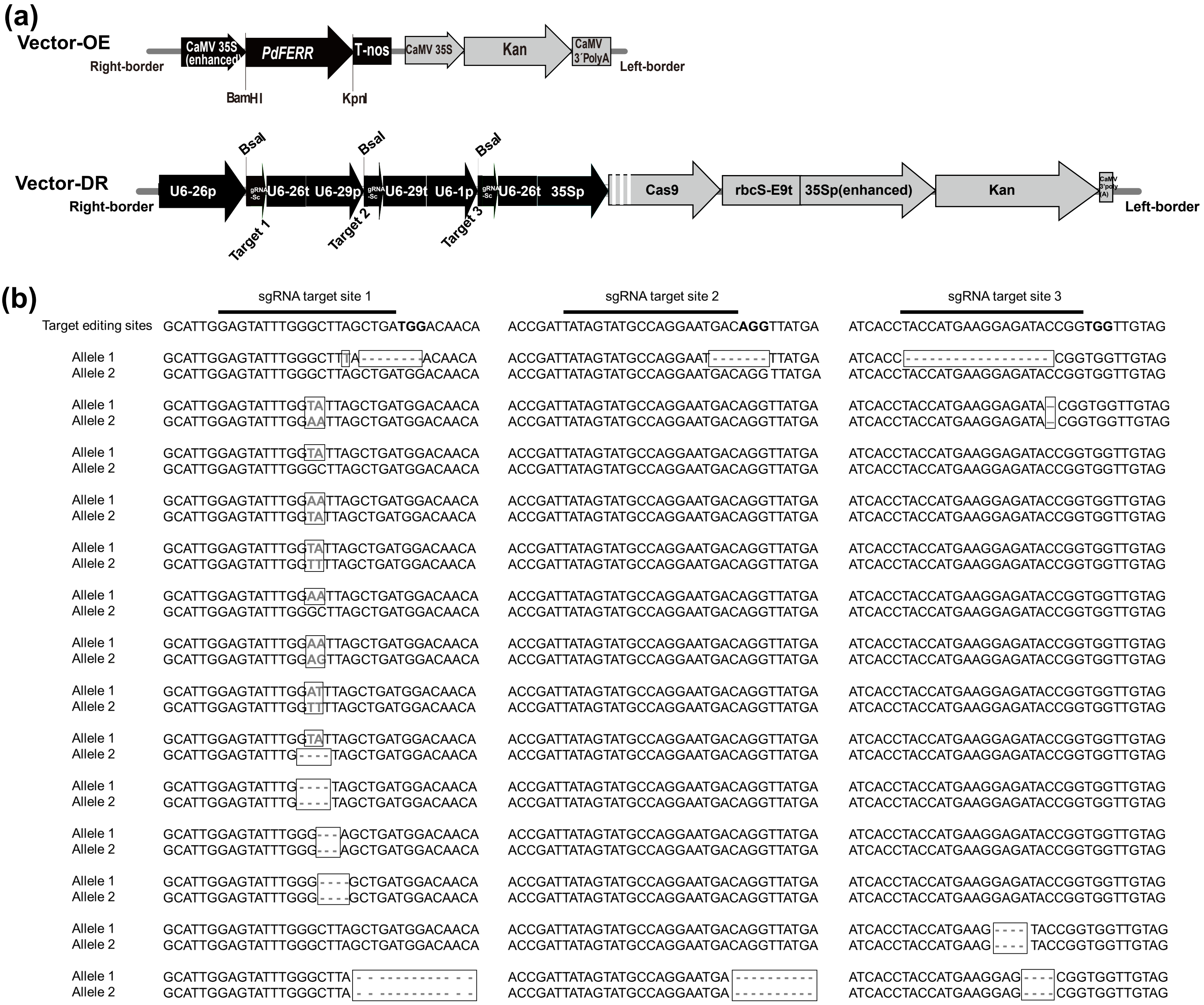
(a)
Vector-OE
Right-border
CaMV 35S (enhanced)
PdFERR
T-nos
CaMV 35S
Kan
CaMV 3´PolyA
Left-border
BamHI
KpnI
Vector-DR
Right-border
BsaI
BsaI
BsaI
U6-26p
gRNA-Sc
U6-26t
U6-29p
gRNA-Sc
U6-29t
U6-1p
gRNA-Sc
U6-26t
35Sp
Cas9
rbcS-E9t
35Sp(enhanced)
Kan
CaMV 3'poly (A)
Left-border
Target 1
Target 2
Target 3
(b)
sgRNA target site 1
sgRNA target site 2
sgRNA target site 3
Target editing sites GCATTGGAGTATTTGGGCTTAGCTGATGGACAACA ACCGATTATAGTATGCCAGGAATGACAGGTTATGA ATCACCTACCATGAAGGAGATACCGGTGGTTGTAG
Allele 1 GCATTGGAGTATTTGGGCTTTA--------ACAACA ACCGATTATAGTATGCCAGGAAT-------TTATGA ATCACC------------------CGGTGGTTGTAG
Allele 2 GCATTGGAGTATTTGGGCTTAGCTGATGGACAACA ACCGATTATAGTATGCCAGGAATGACAGG TTATGA ATCACCTACCATGAAGGAGATACCGGTGGTTGTAG
Allele 1 GCATTGGAGTATTTGGTATTAGCTGATGGACAACA ACCGATTATAGTATGCCAGGAATGACAGGTTATGA ATCACCTACCATGAAGGAGATA- CGGTGGTTGTAG
Allele 2 GCATTGGAGTATTTGGAATTAGCTGATGGACAACA ACCGATTATAGTATGCCAGGAATGACAGGTTATGA ATCACCTACCATGAAGGAGATA- CGGTGGTTGTAG
Allele 1 GCATTGGAGTATTTGGTATTAGCTGATGGACAACA ACCGATTATAGTATGCCAGGAATGACAGGTTATGA ATCACCTACCATGAAGGAGATACCGGTGGTTGTAG
Allele 2 GCATTGGAGTATTTGGGCTTAGCTGATGGACAACA ACCGATTATAGTATGCCAGGAATGACAGGTTATGA ATCACCTACCATGAAGGAGATACCGGTGGTTGTAG
Allele 1 GCATTGGAGTATTTGGAATTAGCTGATGGACAACA ACCGATTATAGTATGCCAGGAATGACAGGTTATGA ATCACCTACCATGAAGGAGATACCGGTGGTTGTAG
Allele 2 GCATTGGAGTATTTGGTATTAGCTGATGGACAACA ACCGATTATAGTATGCCAGGAATGACAGGTTATGA ATCACCTACCATGAAGGAGATACCGGTGGTTGTAG
Allele 1 GCATTGGAGTATTTGGTATTAGCTGATGGACAACA ACCGATTATAGTATGCCAGGAATGACAGGTTATGA ATCACCTACCATGAAGGAGATACCGGTGGTTGTAG
Allele 2 GCATTGGAGTATTTGGTTTTAGCTGATGGACAACA ACCGATTATAGTATGCCAGGAATGACAGGTTATGA ATCACCTACCATGAAGGAGATACCGGTGGTTGTAG
Allele 1 GCATTGGAGTATTTGGAATTAGCTGATGGACAACA ACCGATTATAGTATGCCAGGAATGACAGGTTATGA ATCACCTACCATGAAGGAGATACCGGTGGTTGTAG
Allele 2 GCATTGGAGTATTTGGGCTTAGCTGATGGACAACA ACCGATTATAGTATGCCAGGAATGACAGGTTATGA ATCACCTACCATGAAGGAGATACCGGTGGTTGTAG
Allele 1 GCATTGGAGTATTTGGAATTAGCTGATGGACAACA ACCGATTATAGTATGCCAGGAATGACAGGTTATGA ATCACCTACCATGAAGGAGATACCGGTGGTTGTAG
Allele 2 GCATTGGAGTATTTGGAGTTAGCTGATGGACAACA ACCGATTATAGTATGCCAGGAATGACAGGTTATGA ATCACCTACCATGAAGGAGATACCGGTGGTTGTAG
Allele 1 GCATTGGAGTATTTGGATTTAGCTGATGGACAACA ACCGATTATAGTATGCCAGGAATGACAGGTTATGA ATCACCTACCATGAAGGAGATACCGGTGGTTGTAG
Allele 2 GCATTGGAGTATTTGGTTTTAGCTGATGGACAACA ACCGATTATAGTATGCCAGGAATGACAGGTTATGA ATCACCTACCATGAAGGAGATACCGGTGGTTGTAG
Allele 1 GCATTGGAGTATTTGGTATTAGCTGATGGACAACA ACCGATTATAGTATGCCAGGAATGACAGGTTATGA ATCACCTACCATGAAGGAGATACCGGTGGTTGTAG
Allele 2 GCATTGGAGTATTTG----TAGCTGATGGACAACA ACCGATTATAGTATGCCAGGAATGACAGGTTATGA ATCACCTACCATGAAGGAGATACCGGTGGTTGTAG
Allele 1 GCATTGGAGTATTTG----TAGCTGATGGACAACA ACCGATTATAGTATGCCAGGAATGACAGGTTATGA ATCACCTACCATGAAGGAGATACCGGTGGTTGTAG
Allele 2 GCATTGGAGTATTTG----TAGCTGATGGACAACA ACCGATTATAGTATGCCAGGAATGACAGGTTATGA ATCACCTACCATGAAGGAGATACCGGTGGTTGTAG
Allele 1 GCATTGGAGTATTTGGG---AGCTGATGGACAACA ACCGATTATAGTATGCCAGGAATGACAGGTTATGA ATCACCTACCATGAAGGAGATACCGGTGGTTGTAG
Allele 2 GCATTGGAGTATTTGGG---AGCTGATGGACAACA ACCGATTATAGTATGCCAGGAATGACAGGTTATGA ATCACCTACCATGAAGGAGATACCGGTGGTTGTAG
Allele 1 GCATTGGAGTATTTGGG----GCTGATGGACAACA ACCGATTATAGTATGCCAGGAATGACAGGTTATGA ATCACCTACCATGAAGGAGATACCGGTGGTTGTAG
Allele 2 GCATTGGAGTATTTGGG----GCTGATGGACAACA ACCGATTATAGTATGCCAGGAATGACAGGTTATGA ATCACCTACCATGAAGGAGATACCGGTGGTTGTAG
Allele 1 GCATTGGAGTATTTGGGCTTAGCTGATGGACAACA ACCGATTATAGTATGCCAGGAATGACAGGTTATGA ATCACCTACCATGAAG---- TACCGGTGGTTGTAG
Allele 2 GCATTGGAGTATTTGGGCTTAGCTGATGGACAACA ACCGATTATAGTATGCCAGGAATGACAGGTTATGA ATCACCTACCATGAAG---- TACCGGTGGTTGTAG
Allele 1 GCATTGGAGTATTTGGGCTTA- - ----------- - ACCGATTATAGTATGCCAGGAATGA---------- ATCACCTACCATGAAGGAG----CGGTGGTTGTAG
Allele 2 GCATTGGAGTATTTGGGCTTA- - ----------- - ACCGATTATAGTATGCCAGGAATGA---------- ATCACCTACCATGAAGGAG----CGGTGGTTGTAG
