# Supplementary material for: Expression of poplar sex-determining gene affects plant drought tolerance and the underlying molecular mechanism
Source: Hortic Res. 2025 Mar 5;12(6):uhaf066. doi: 10.1093/hr/uhaf066 (PMC12038252; doi:10.1093/hr/uhaf066)
Supplement: Web_Material_uhaf066 [file web_material_uhaf066.zip › Supplementary Figure_Marked.docx]

**Supplementary Figure**

**
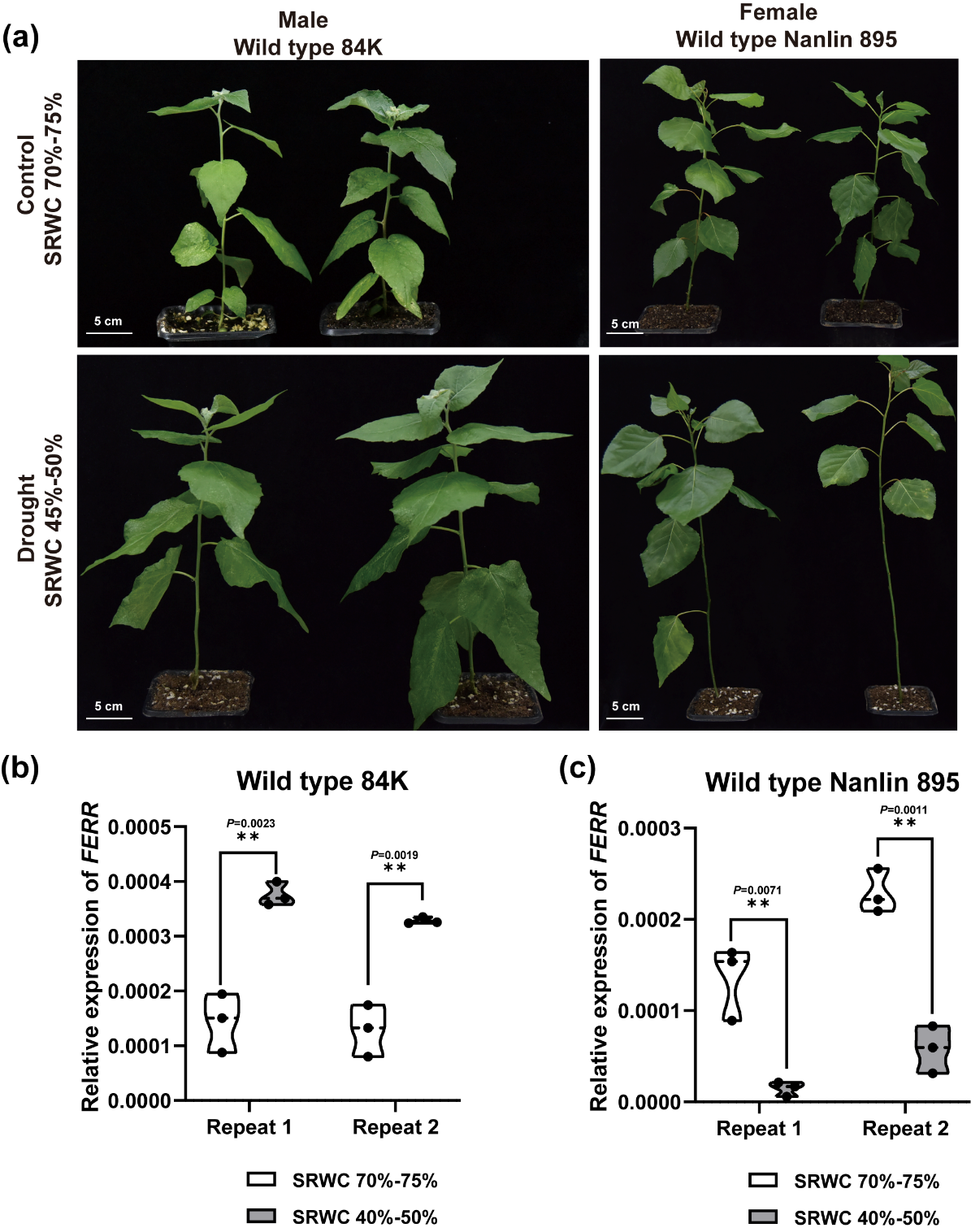
**

**Figure S1.** *FERR* expression influences drought tolerance. **(a)** Photographs of the wild type male poplar (“84K”) and female poplar (“Nanlin 895”) under well-watered (70%-75% SRWC) and moderate drought conditions (40%-50% SRWC). Before photography, plants cultivated for 60 days were subjected to drought stress by withholding water for 7 days. **(b)** Expression of *FERR* in wild type “84K”. **(c)** Expression of *FERR* in wild type “Nanlin 895”. Asterisks indicate significant differences from the wild-type (**P* < 0.05; ***P* < 0.01; two-sided Student’s *t*-test). qRT-PCR analysis with two biological replicates and three technical replicates. SRWC: soil relative water content.

**
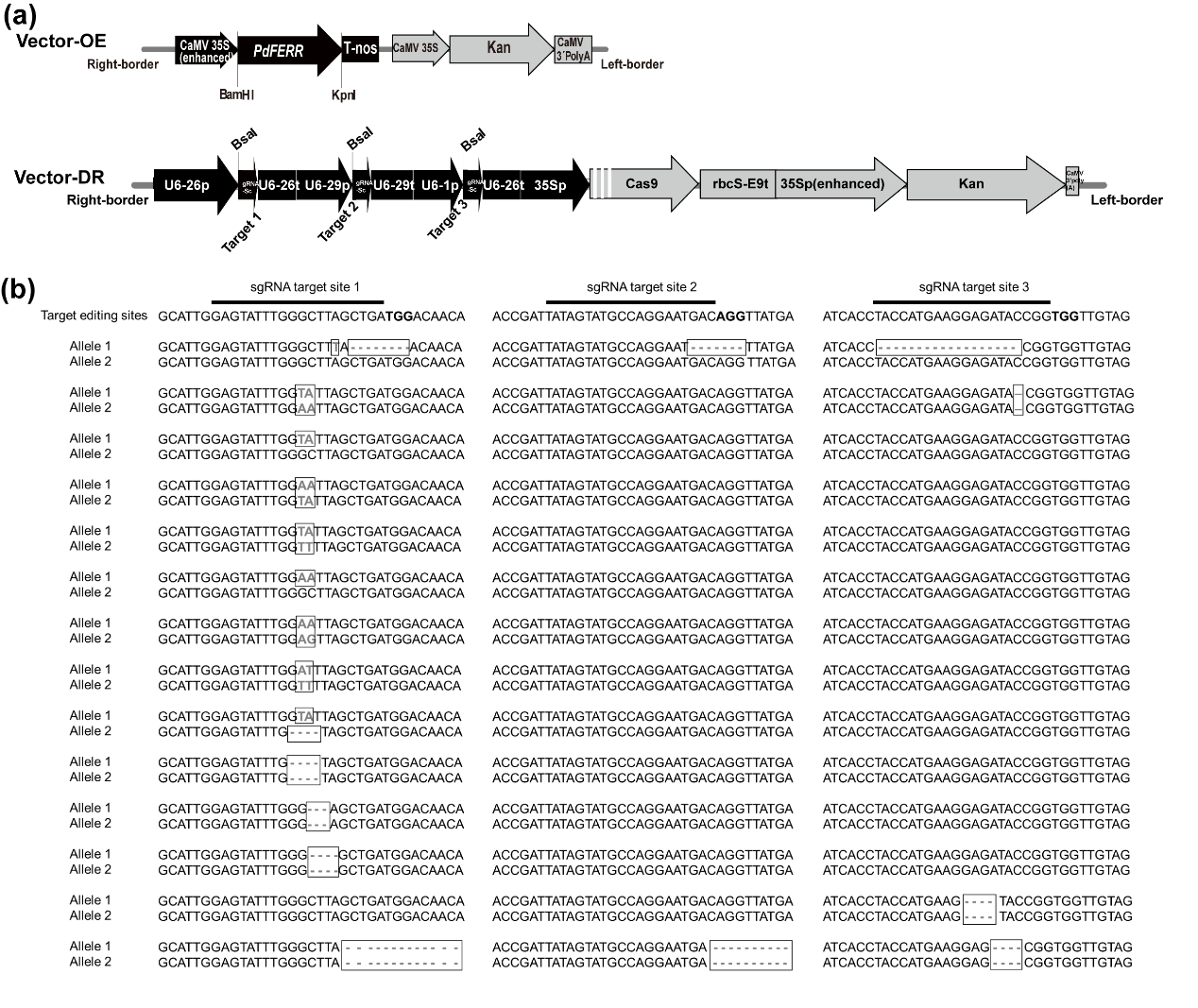
**

**Figure S2.** Schematic diagram of vector structure and the gene editing types. **(a)** The upper panel shows the schematic diagram of the over-expression vector, and the lower panel shows the schematic diagram of the CRISPR/Cas9 knockout vector. **(b)** Editing types for DNA fragments containing three targeting sites. The wild-type sequence is shown at the top with three target sites highlighted with horizontal lines and the PAM sequence highlighted in bold. Dashes indicate nucleotide deletions.


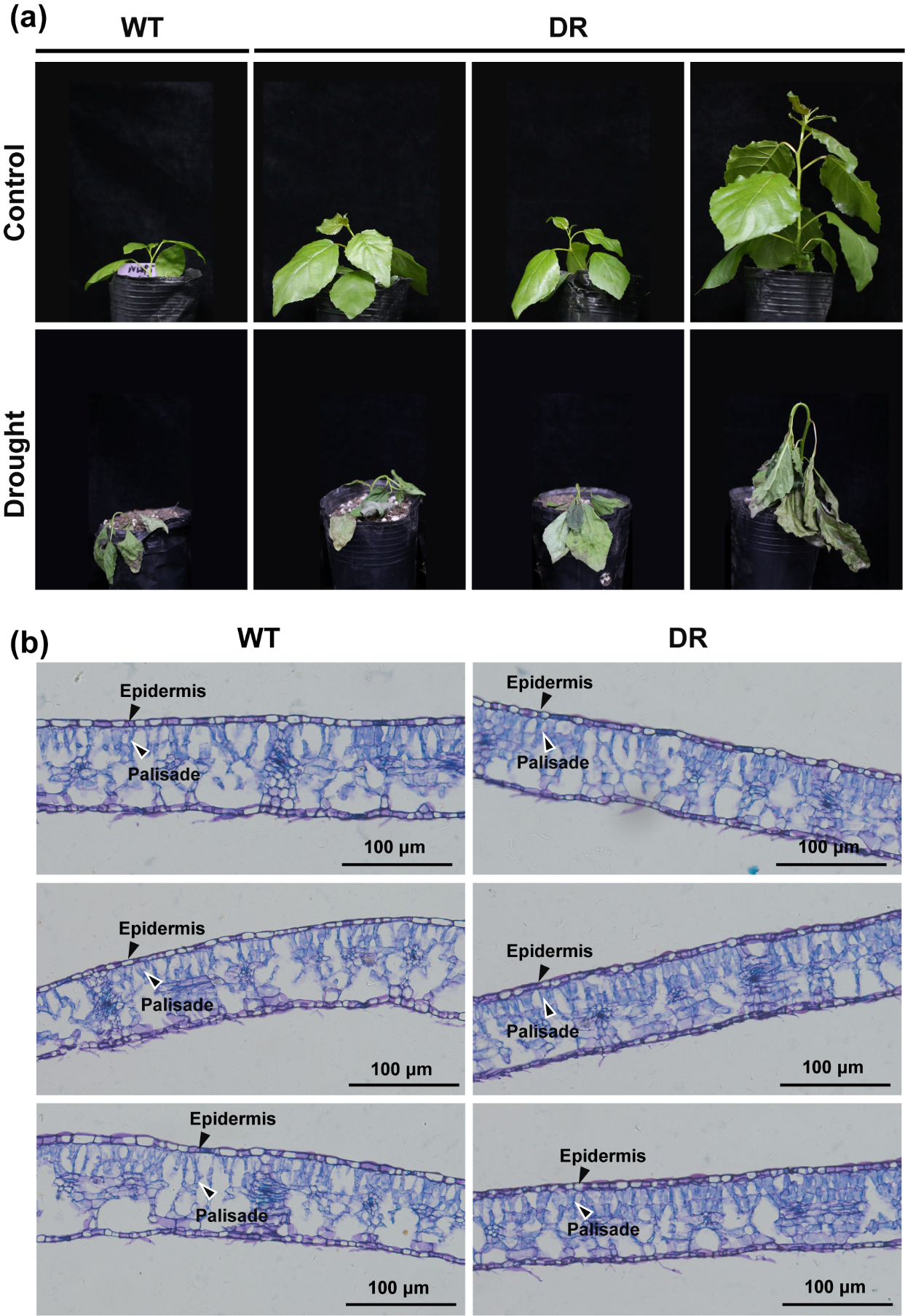


**Figure S3.** Phenotypic comparison of knockout lines under drought stress. **(a)** Comparison of drought resistance between knockout lines and the wild type of “Nanlin 895” under well-watered and drought stress conditions. **(b)** Structural analysis of the leaf palisade and epidermis showing no significant changes.

**
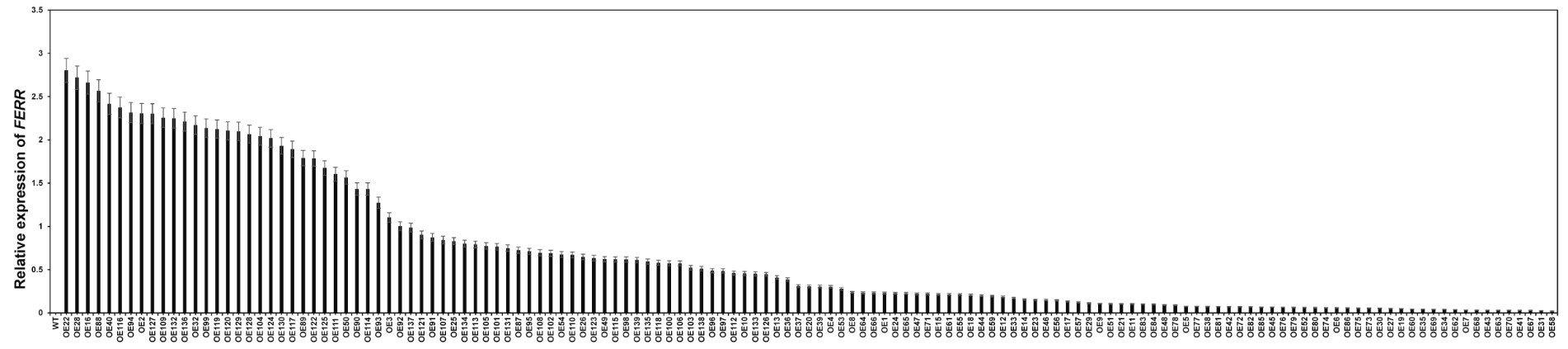
**

**Figure S4.** Relative expression levels of *FERR* in the 139 positive over-expression lines.

**
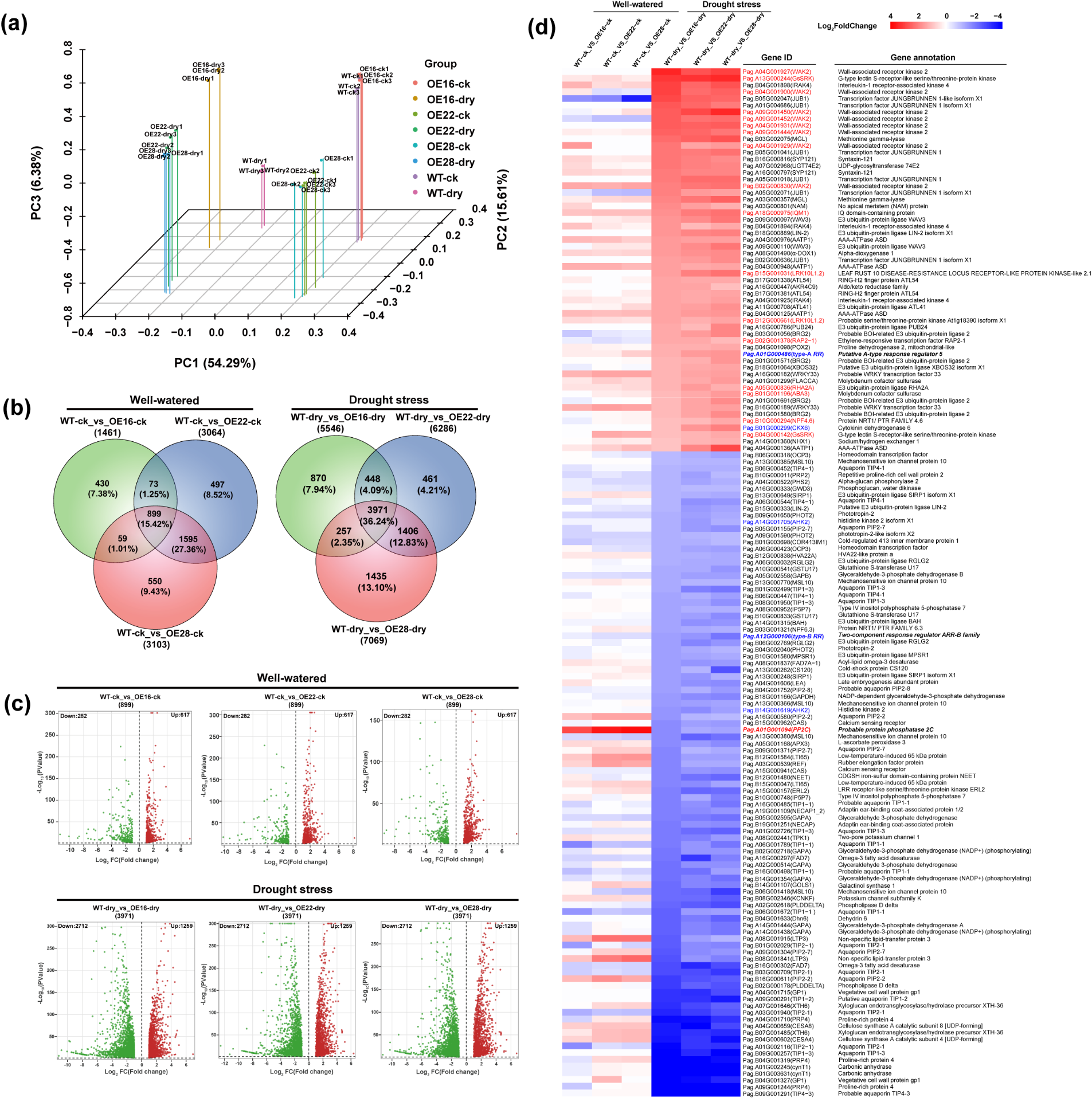
**

**Figure S5.** RNA-seq data quality, differential expression analysis, and functional annotation of drought-responsive genes. **(a)** PCA plot showing clear clustering of samples by treatment and genotype. **(b)** Venn diagrams showing the number and overlap of differentially expressed genes (DEGs) in transgenic (OE) plants compared to WT under well-watered and drought conditions. Significant DEGs are visualized (red: upregulated, green: downregulated). **(c)** Volcano plots depicting DEG distribution for each comparison. **(d)** Functional annotation of known drought-related genes within molecular modules MEturquoise, MEyellow, MEblack, and MEblue. Gene IDs in blue are the up-regulated and down-regulated DEGs involved in cytokinin signaling, and those in red are the up-regulated and down-regulated DEGs involved in ABA signaling. Among these DEGs, the key genes involved in crosstalk between cytokinin and ABA signaling were shown in italic bold font.

**
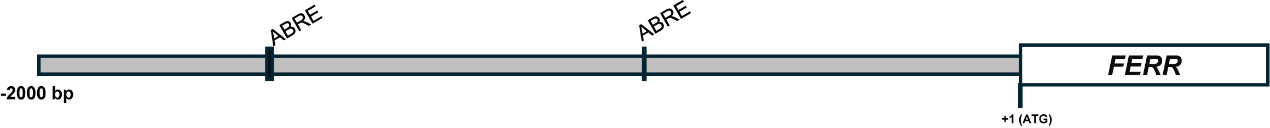
**

**Figure S6. The locations of ABA response elements (ABRE) in the 5’ flanking region of the *FERR* gene.**


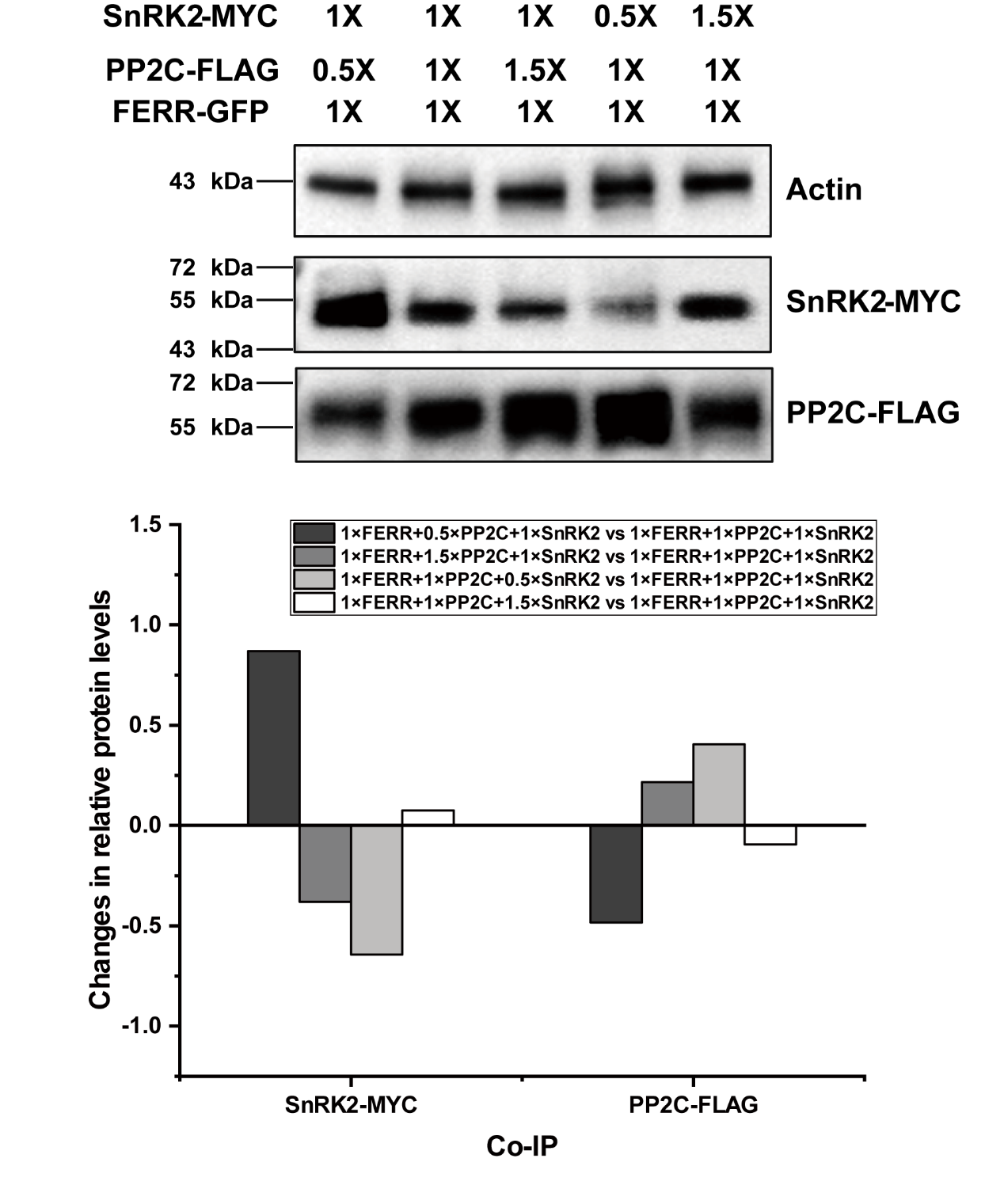


**Figure S7.** Quantification of western blot analysis images. Graphical representation of western blot quantification comparing protein levels was generated using ImageJ software under different combinations of FERR, PP2C, and SnRK2. The following conditions were compared to the 1 x FERR+1 x PP2C+1 x SnRK2 level in each sample: 1 x FERR+0.5 x PP2C+1 x SnRK2, 1 x FERR+1.5 x PP2C+1 x SnRK2, 1 x FERR+1 x PP2C+0.5 x SnRK2, and 1 x FERR+1 x PP2C+1.5 x SnRK2.
