# Supplementary material for: Expression of poplar sex-determining gene affects plant drought tolerance and the underlying molecular mechanism
Source: Hortic Res. 2025 Mar 5;12(6):uhaf066. doi: 10.1093/hr/uhaf066 (PMC12038252; doi:10.1093/hr/uhaf066)
Supplement: Web_Material_uhaf066 [file web_material_uhaf066.zip › Supplementary Table Marked.docx]

**Supplementary Table**

**Table S1** Primers in this study.

| **Application** | **Primer sequence (5′-3′)** |
| --- | --- |
| *FERR* amplification | GTGTTTTGTTGAGGAGATTAGAC (forward) |
|  | CCCTTCTTGTCTCTCTTTCTG (reverse) |
| Construction of 35S: *FERR*- OE vector | GACTCTAGAGGATCCATGGCCAGCTCTTCTTCTTCC (forward) |
|  | GGAAATTCGAGCTCGGTACCTTATGCATGTTCGTCTTCTCCTGCAA (reverse) |
| *Kana* gene amplification | CTGAAGCGGGAAGGGACT (forward) |
|  | ATACCGTAAAGCACGAGGAAG (reverse) |
| Reference gene (*UBIQUITIN*, *UBQ*) qRT-PCR | GTTGATTTTTGCTGGGAAGC (forward) |
|  | GATCTTGGCCTTCACGTTGT (reverse) |
| *FERR* gene qRT-PCR | AAACGGAAAGAGAGCATTGGA (forward) |
|  | TCATAACCTGTCATTCCTGGCA (reverse) |
| T1 and T2 amplification | ACCACAGCAGAAAACGG (forward) |
|  | AAGGACTCCCAAAAAAT (reverse) |
| T3 amplification | GAATGTCCAGTAATTAAGAGAGCATCG (forward) |
|  | GACTGAAATTGCTGAGCAGG (reverse) |
| T1, T2, and T3 amplification | GTGGCAGAAGGTTAGACT (forward) |
|  | TGAAATTGCTGAGCAGGT (reverse) |
| *ARR5* gene qRT-PCR | TGTGGAAATGGCTCTGGCTA |
|  | TCCACTGCCGTGACTTGATA |
| *PagBRR13* gene qRT-PCR | TGCAGCTTGGTCTTGGAAAC |
|  | CCCAAAGCATGTCGCTAACA |
| *PP2C* gene qRT-PCR | GTGGTGGTGATGGTAGGGAA |
|  | GCTGCTTCCACTCTCTCTCT |
| *SnRK2* gene qRT-PCR | GAGAGGTACGAGCTGGTGAA |
|  | GAGAGGTACGAGCTGGTGAA |
| Construction of FERR-pGBKT7 vector | AGGAGGACCTGCATATGATGGCCAGCTCTTCTTCTTCC (forward) |
|  | GCCGCTGCAGGTCGACTTATGCATGTTCGTCTTCTCCTGC (reverse) |
| Construction of pGADT7-SnRK2 vector | GCCATGGAGGCCAGTGAATTATGGAGAGGTACGAGCTGGTGAAAG (forward) |
|  | CAGCTCGAGCTCGATGGATCTTAACTGACATGAACTTCCCCGCTTGC (reverse) |
| Construction of SnRK2- pGBKT7 vector | ATGGCCATGGAGGCCGAATTATGGAGAGGTACGAGCTGGTGAAAG (forward) |
|  | CCGCTGCAGGTCGACGGATCTTAACTGACATGAACTTCCCCGCTTGC (reverse) |
| Construction of pGADT7-ARR5 vector | GCCATGGAGGCCAGTGAATTATGGCTGTGGAAATGGCTCTGGC (forward) |
|  | CAGCTCGAGCTCGATGGATCTCAGACAACTTCCAAGCCATCATTGTATCTTG (reverse) |
| Construction of FERR-cYFP vector | ATTACAGGTACCCGGGGATCATGGCCAGCTCTTCTTCTTCCTTACC (forward) |
|  | GCCACCGCCGTCGACTCTAGTGCATGTTCGTCTTCTCCTGCAAAGC (reverse) |
| Construction of SnRK2-nYFP vector | ATCGAGGACGCCGGCGGATCATGGAGAGGTACGAGCTGGTGAAAG (forward) |
|  | GCTCTGCAGGTCGACTCTAGTTAACTGACATGAACTTCCCCGCTTGC (reverse) |
| Construction of ARR5–cYFP vector | ATTACAGGTACCCGGGGATCATGGCTGTGGAAATGGCTCTGGC (forward) |
|  | GCCACCGCCGTCGACTCTAGGACAACTTCCAAGCCATCATTGTATCTTGTTC (reverse) |
| Construction of FERR-cLUC vector | TACGCGTCCCGGGGCGGTACATGGCCAGCTCTTCTTCTTCCTTACC (forward) |
|  | ACGAAAGCTCTGCAGGTCGATTATGCATGTTCGTCTTCTCCTGCAAAGC (reverse) |
| Construction of SnRK2-nLUC vector | CGAGCTCGGTACCCGGGATCATGGAGAGGTACGAGCTGGTGAAAG forward) |
|  | CGCGTACGAGATCTGGTCGAACTGACATGAACTTCCCCGCTTGC (reverse) |
| Construction of ARR5-cLUC vector | TACGCGTCCCGGGGCGGTACATGGCTGTGGAAATGGCTCTGGC (forward) |
|  | ACGAAAGCTCTGCAGGTCGATCAGACAACTTCCAAGCCATCATTGTATCTTG (reverse) |
| Construction of pMAL-c5x-FERR vector | GGGAAGGATTTCACATATGATGGCAAGCAGCAGCAGCTC (forward) |
|  | AATTACCTGCAGGGAATTCTTATGCATGTTCATCTTCACC (reverse) |
| Construction of pGEX-6p-1-SnRK2 vector | TTCCAGGGGCCCCTGGGATCATGGAGAGGTACGAGCTGGTGAAAG (forward) |
|  | GTCACGATGCGGCCGCTCGATTAACTGACATGAACTTCCCCGCTTGC (reverse) |
| Construction of pET28a-ARR5 vector | ATGGGTCGCGGATCCGAATTATGGCTGTGGAAATGGCTCTGGC (forward) |
|  | GTGGTGGTGGTGGTGCTCGAGACAACTTCCAAGCCATCATTGTATCTTGTTC (reverse) |
| Construction of PP2C-FLAG vector | ACATTTACAATTACGGATCCATGATGGCTTCTGCTGCTACGTATTCATTC (forward) |
|  | CCGTCGTGGTCTTTGTAATCGCTTTTTTTCAGCTGAACTACTATGACACTGATATTG (reverse) |
| Construction of SnRK2-MYC vector | ACATTTACAATTACGGATCCATGGAGAGGTACGAGCTGGTGAAAG (forward) |
|  | TCACCGTTAATCAAACCCATACTGACATGAACTTCCCCGCTTGC (reverse) |
| Construction of FERR-GFP vector | GGGGACAAGTTTGTACAAAAAAGCAGGCTTAATGGCCAGCTCTTCTTCTTCC (forward) |
|  | GGACTCTAGAGGATCCATGGCCAGCTCTTCTTCTTCC (reverse) |
| **Target sites** | **Target sequence (5′-3′)** |
| sgRNA1 | GAGTATTTGGGCTTAGCTGA TGG (45%, 0.333) |
| sgRNA2 | TATAGTATGCCAGGAATGAC AGG (40%, 0.273) |
| sgRNA3 | TACCATGAAGGAGATACCGG TGG (50%, 0.342) |

**Table S2** Effect of *FERR* on root biomass in the knockout lines.

| **Root biomass** | **Knockout lines** | | | | | **Wild type plants** | | | | | ***t*-test** |
| --- | --- | --- | --- | --- | --- | --- | --- | --- | --- | --- | --- |
|  | ***n*** | **Mean**  **(g)** | **SD.** | **Range** | **CV** | ***n*** | **Mean**  **(g)** | **SD.** | **Range** | **CV** | ***P*** |
| Fresh weight | 15 | 3.29 | 0.50 | 2.18-3.91 | 0.15 | 15 | 3.16 | 0.28 | 2.32-3.72 | 0.09 | 0.4040 |
| Dry weight | 15 | 0.54 | 0.16 | 0.31-0.90 | 0.30 | 15 | 0.60 | 0.09 | 0.49-0.79 | 0.14 | 0.1467 |

**Table S3** Effect of *FERR* on relative water content (%) in leaves of the knockout lines.

| **Treatment** | **Knockout lines** | | | | | **Wild type plants** | | | | | ***t*-test** |
| --- | --- | --- | --- | --- | --- | --- | --- | --- | --- | --- | --- |
|  | ***n*** | **Mean** | **SD.** | **Range** | **CV** | ***n*** | **Mean** | **SD.** | **Range** | **CV** | ***P*** |
| Control | 27 | 91.48 | 1.21 | 89.37-93.27 | 0.01 | 9 | 88.34 | 0.81 | 86.77-89.90 | 0.01 | 0.0982 |
| Drought | 27 | 42.97 | 1.49 | 41.32-45.48 | 0.03 | 9 | 41.00 | 0.47 | 40.26-42.02 | 0.01 | 0.1045 |

**Table S4** Summary of sample sequencing data quality.

| **Sample** | **ReadSum** | **BaseSum** | **GC (%)** | **N (%)** | **Q20 (%)** | **CycleQ20 (%)** | **Q30 (%)** |
| --- | --- | --- | --- | --- | --- | --- | --- |
| OE16-ck1 | 25759381 | 7727814300 | 43.83 | 0 | 95.69 | 100 | 89.82 |
| OE16-ck2 | 23491207 | 7047362100 | 43.84 | 0 | 95.6 | 100 | 89.5 |
| OE16-ck3 | 22855629 | 6856688700 | 43.89 | 0 | 96.02 | 100 | 90.25 |
| OE16-dry1 | 24731763 | 7419528900 | 43.98 | 0 | 95.55 | 100 | 89.42 |
| OE16-dry2 | 19598465 | 5879539500 | 43.94 | 0 | 95.07 | 100 | 88.57 |
| OE16-dry3 | 26762146 | 8028643800 | 44.06 | 0 | 96.37 | 100 | 90.9 |
| OE22-ck.1 | 23401159 | 7020347700 | 44.26 | 0 | 97.29 | 100 | 92.38 |
| OE22-ck2 | 23702533 | 7110759900 | 44.12 | 0 | 97.35 | 100 | 92.46 |
| OE22-ck3 | 23782576 | 7134772800 | 44.2 | 0 | 97.7 | 100 | 93.3 |
| OE22-dry1 | 21378127 | 6413438100 | 43.61 | 0 | 97.46 | 100 | 92.71 |
| OE22-dry2 | 24794875 | 7438462500 | 43.93 | 0 | 97.26 | 100 | 92.31 |
| OE22-dry.3 | 25329654 | 7598896200 | 43.87 | 0 | 97.29 | 100 | 92.38 |
| OE28-ck1 | 26921156 | 8076346800 | 43.64 | 0 | 97.45 | 100 | 92.73 |
| OE28-ck2 | 28550653 | 8565195900 | 44.48 | 0 | 97.21 | 100 | 92.26 |
| OE28-ck3 | 22989577 | 6896873100 | 44.31 | 0 | 97.37 | 100 | 92.51 |
| OE28-dry1 | 21672481 | 6501744300 | 43.65 | 0 | 97.65 | 100 | 93.16 |
| OE28-dry2 | 22623384 | 6787015200 | 43.88 | 0 | 97.39 | 100 | 92.58 |
| OE28-dry3 | 29708943 | 8912682900 | 43.88 | 0 | 97.39 | 100 | 92.57 |
| WT-ck1 | 25853518 | 7756055400 | 43.89 | 0 | 95.66 | 100 | 89.61 |
| WT-ck2 | 23578878 | 7073663400 | 43.81 | 0 | 95.96 | 100 | 90.3 |
| WT-ck3 | 21507922 | 6452376600 | 43.98 | 0 | 96.57 | 100 | 91.36 |
| WT-dry1 | 23493869 | 7048160700 | 44.06 | 0 | 96.19 | 100 | 90.63 |
| WT-dry2 | 22003827 | 6601148100 | 44.29 | 0 | 96.33 | 100 | 90.85 |
| WT-dry3 | 23244140 | 6973242000 | 43.98 | 0 | 96.32 | 100 | 90.72 |

**Table S5** Comparison of the expression of *Pag.B04G002139* and *Pag.A04G002172* in the over-expression and the wild type plants.

| **Plant** | ***Pag.B04G002139***  **(FPKM)** | ***Pag.A04G002172***  **(FPKM)** |
| --- | --- | --- |
| WT-dry1 | 10.7349 | 14.2469 |
| WT-dry2 | 11.2812 | 13.4980 |
| WT-dry3 | 12.2108 | 15.3546 |
| Mean | 11.4090 | 14.3665 |
| OE16-dry1 | 11.7202 | 13.7967 |
| OE16-dry2 | 10.8632 | 15.4735 |
| OE16-dry3 | 12.0630 | 14.5481 |
| Mean | 11.5488 | 14.3728 |
| OE22-dry1 | 12.6339 | 15.1508 |
| OE22-dry2 | 13.5882 | 16.4457 |
| OE22-dry3 | 14.7489 | 16.8717 |
| Mean | 13.6570 | 16.1561 |
| OE28-dry1 | 13.9171 | 16.9056 |
| OE28-dry2 | 13.4471 | 18.0127 |
| OE28-dry3 | 14.0764 | 16.1840 |
| Mean | 13.8135 | 17.0341 |
