# Supplementary figures and images for: Expression of poplar sex-determining gene affects plant drought tolerance and the underlying molecular mechanism
Source: Hortic Res. 2025 Mar 5;12(6):uhaf066. doi: 10.1093/hr/uhaf066 (PMC12038252; doi:10.1093/hr/uhaf066)

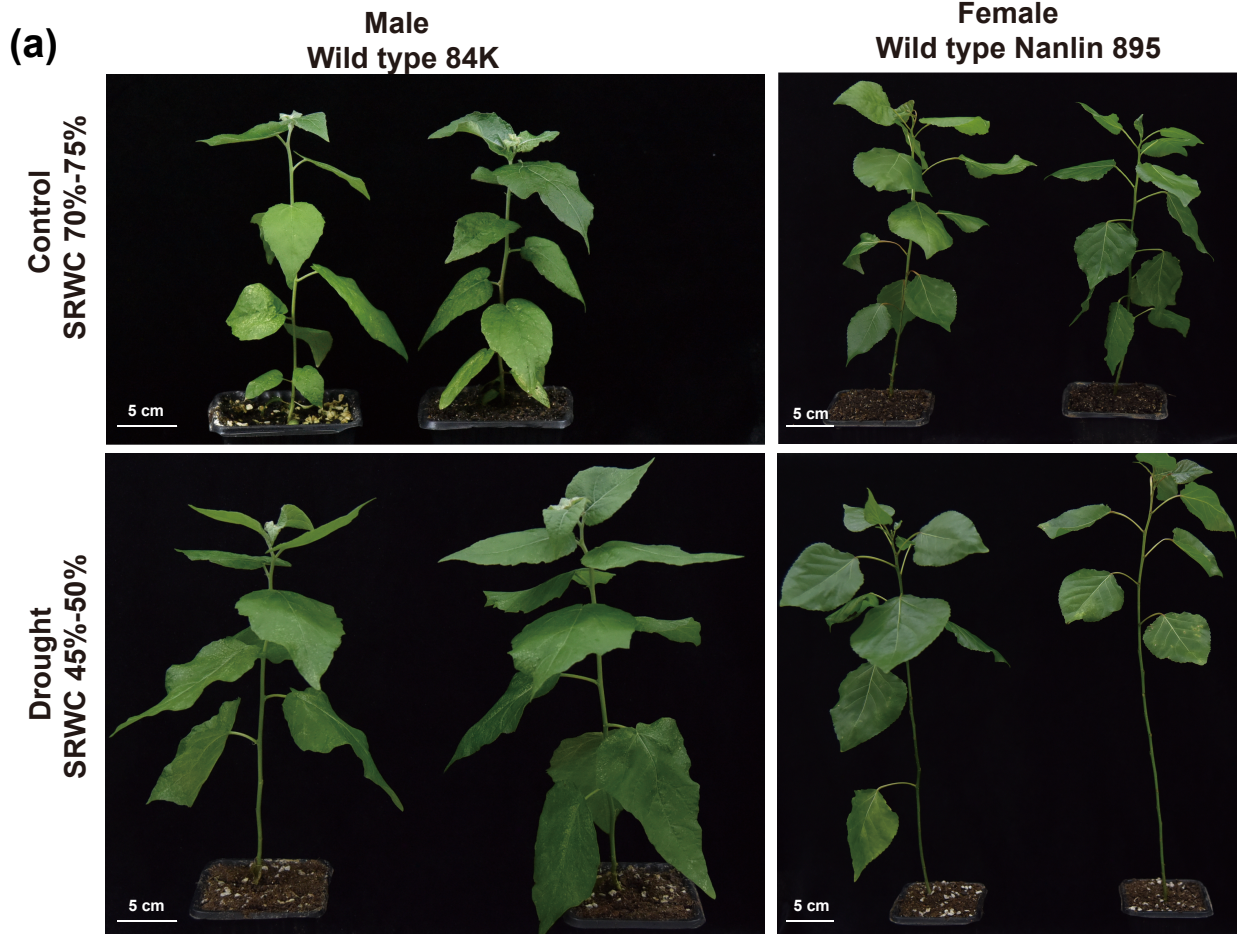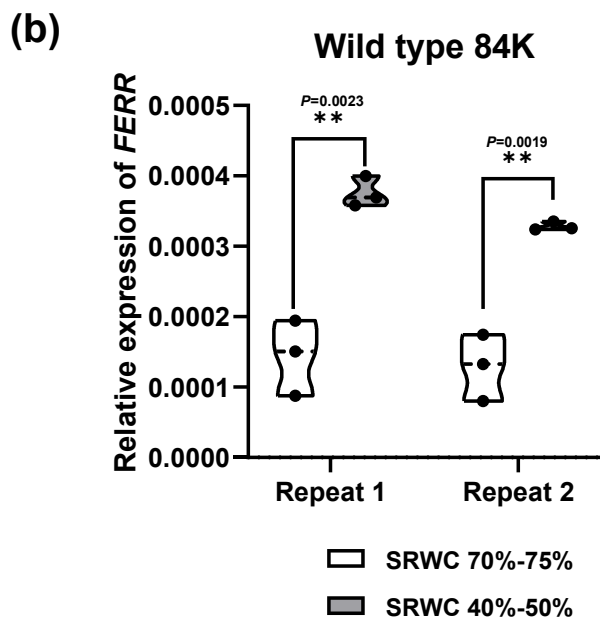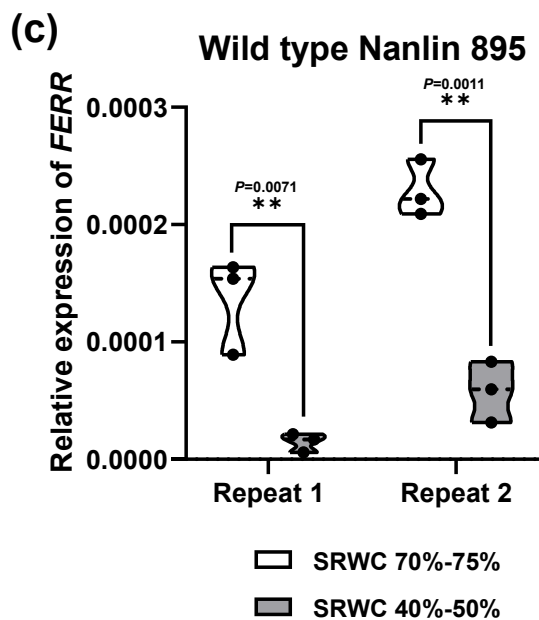

Supplement: Web_Material_uhaf066 [file web_material_uhaf066.zip › Figure S1.pdf]

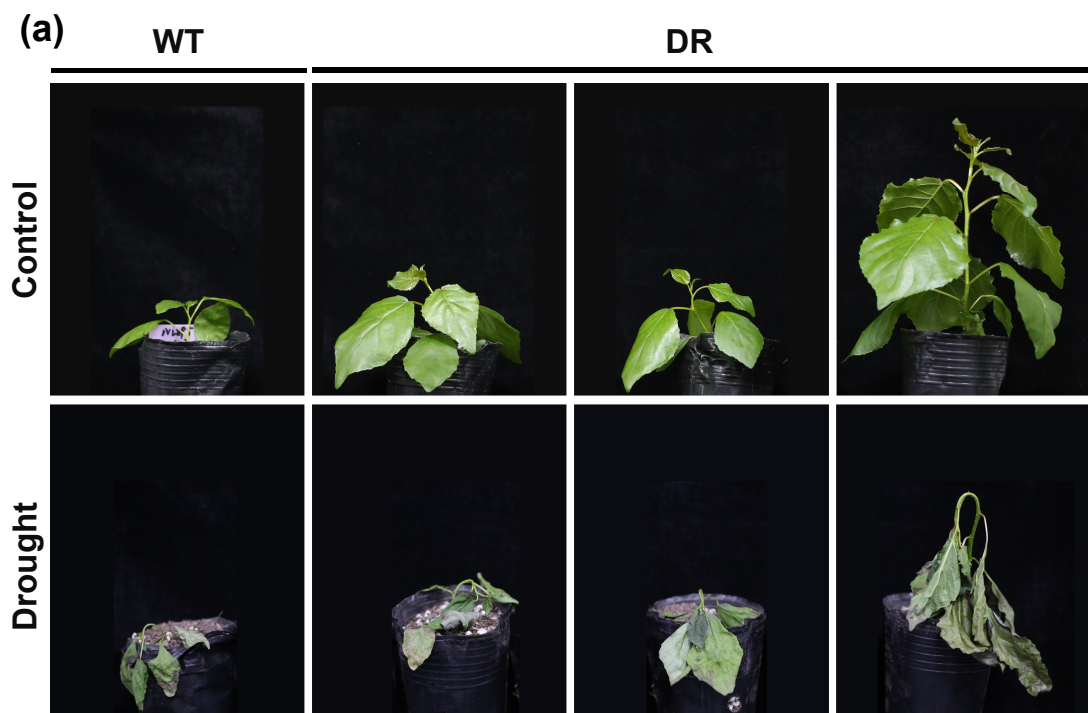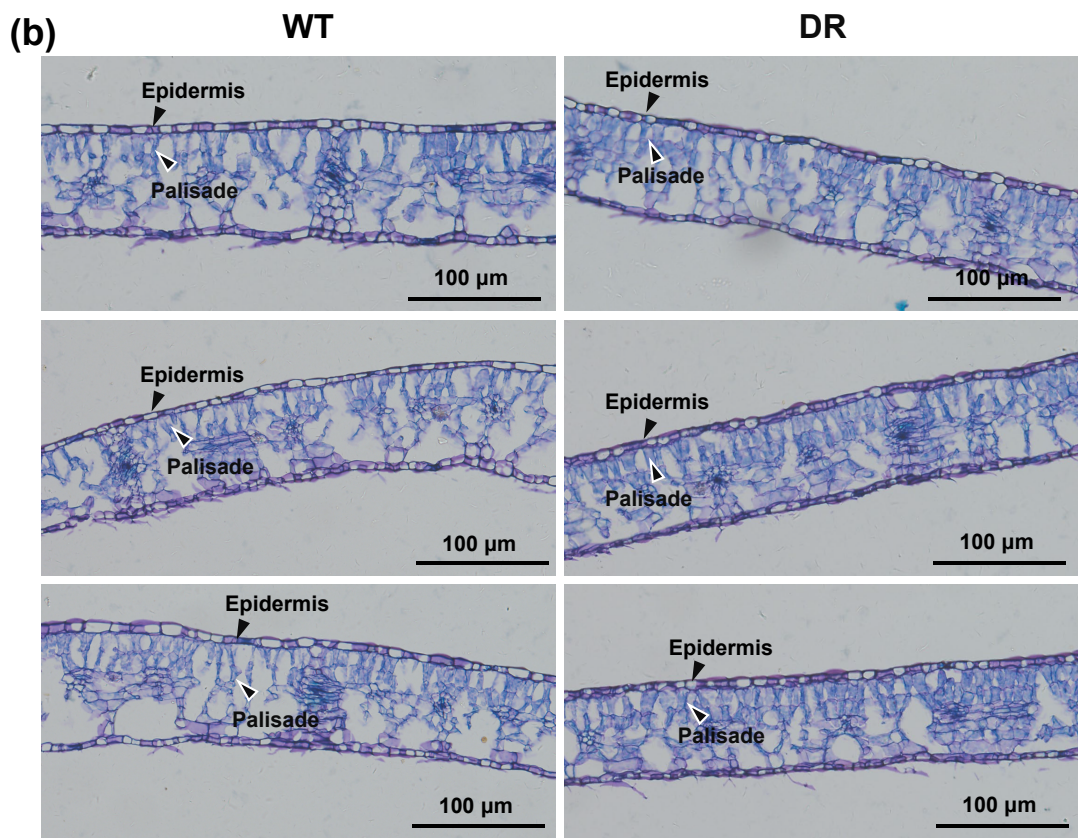

Supplement: Web_Material_uhaf066 [file web_material_uhaf066.zip › Figure S3.pdf]

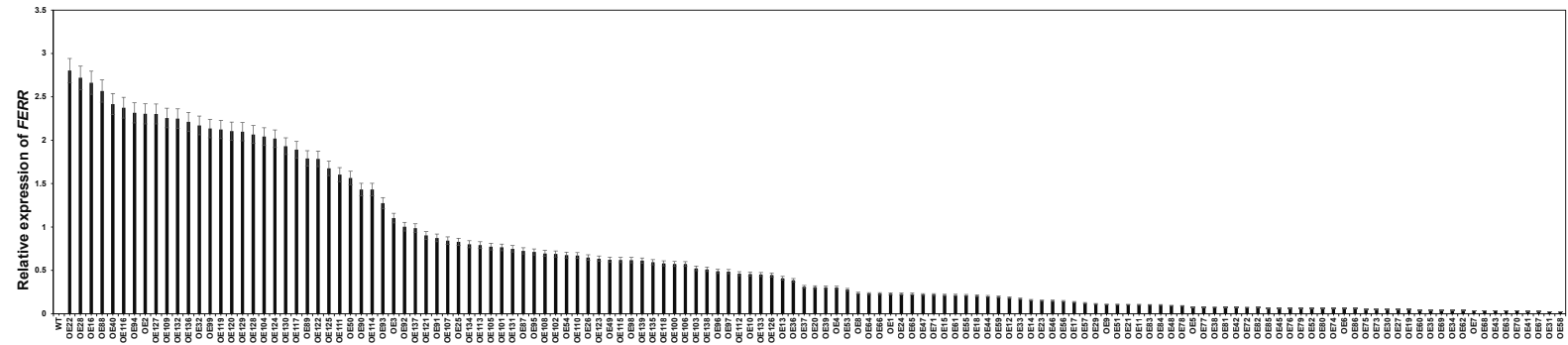

Supplement: Web_Material_uhaf066 [file web_material_uhaf066.zip › Figure S4.pdf]

(a)

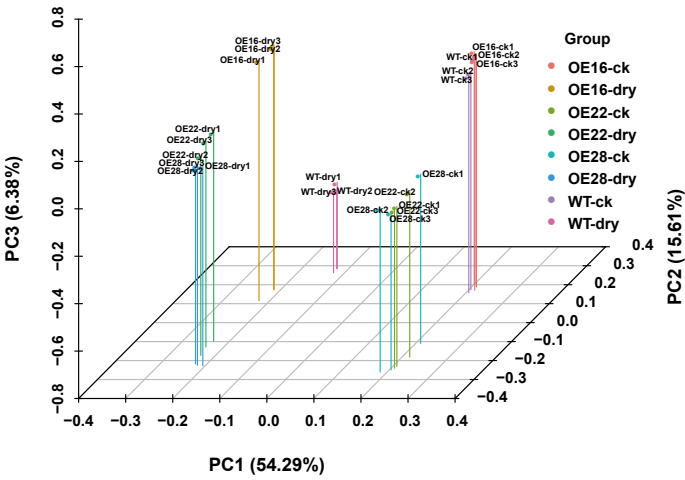

(b)

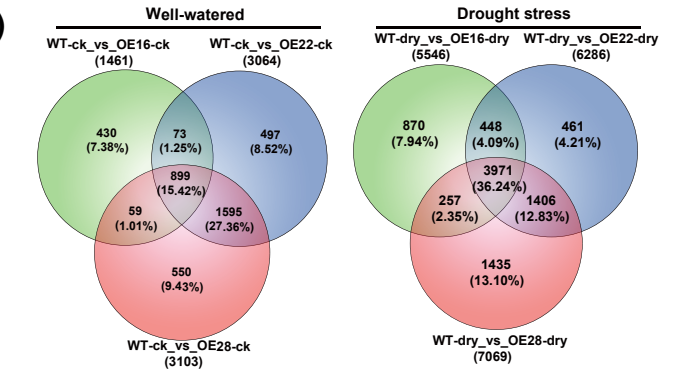

(c)

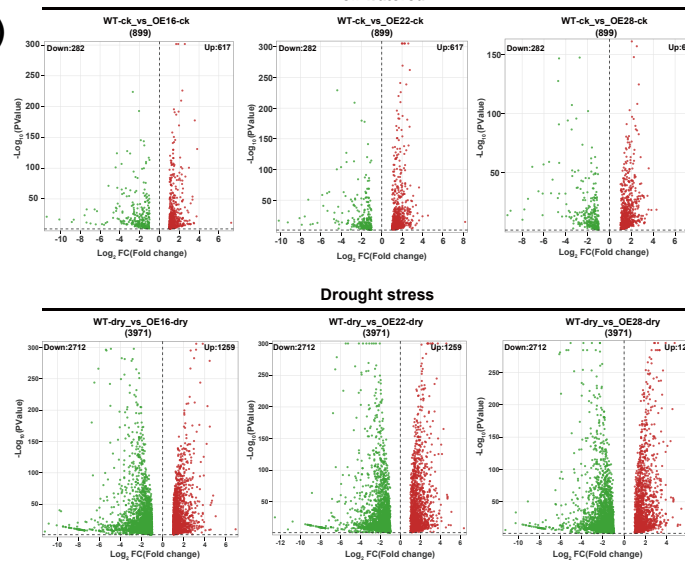

(d)

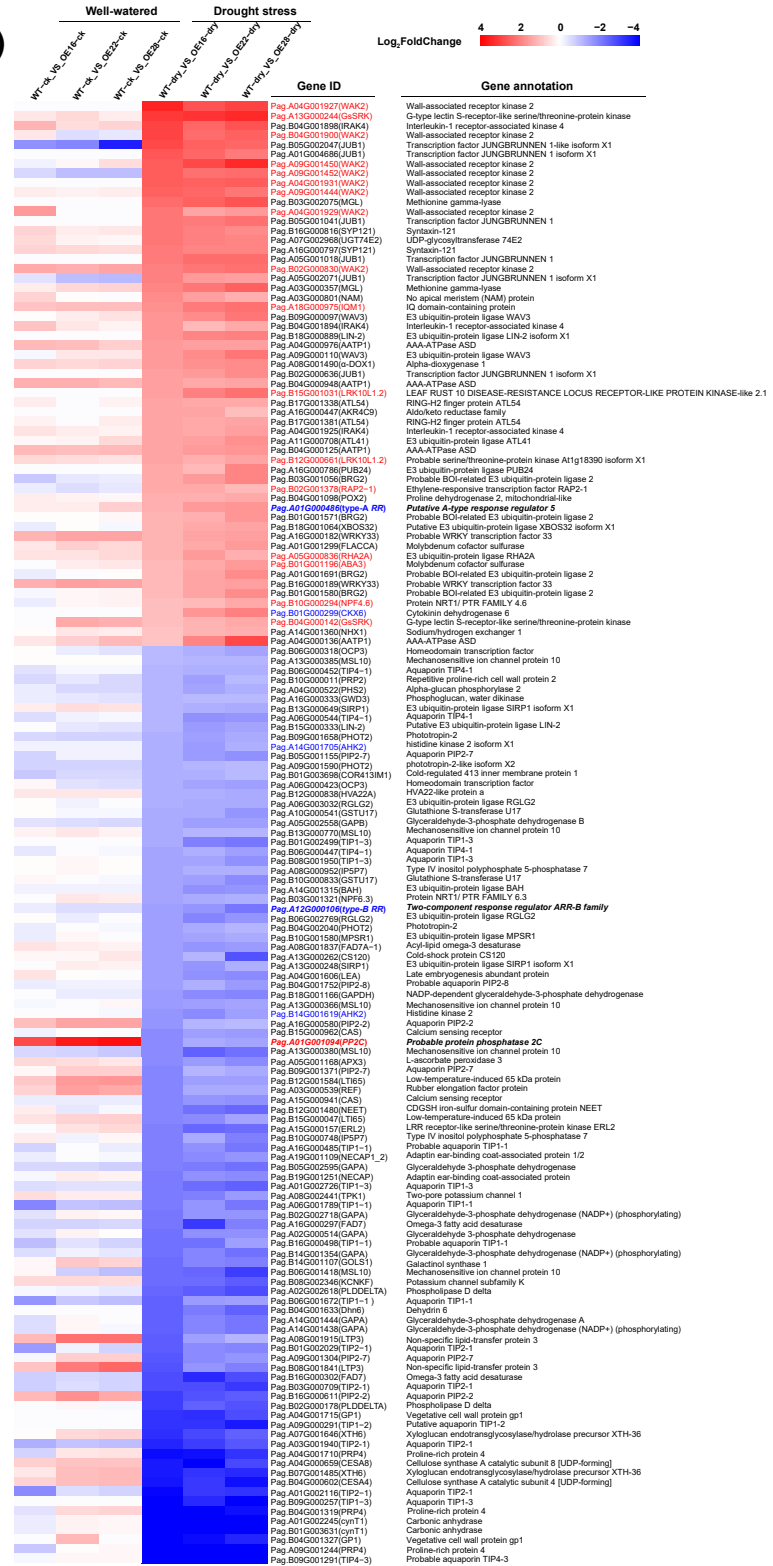

Supplement: Web_Material_uhaf066 [file web_material_uhaf066.zip › Figure S5.pdf]

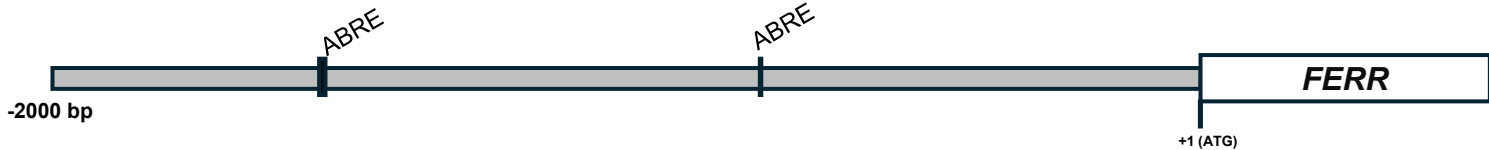

Supplement: Web_Material_uhaf066 [file web_material_uhaf066.zip › Figure S6.pdf]

|           |      |    |      |      |      |
|-----------|------|----|------|------|------|
| SnRK2-MYC | 1X   | 1X | 1X   | 0.5X | 1.5X |
| PP2C-FLAG | 0.5X | 1X | 1.5X | 1X   | 1X   |
| FERR-GFP  | 1X   | 1X | 1X   | 1X   | 1X   |

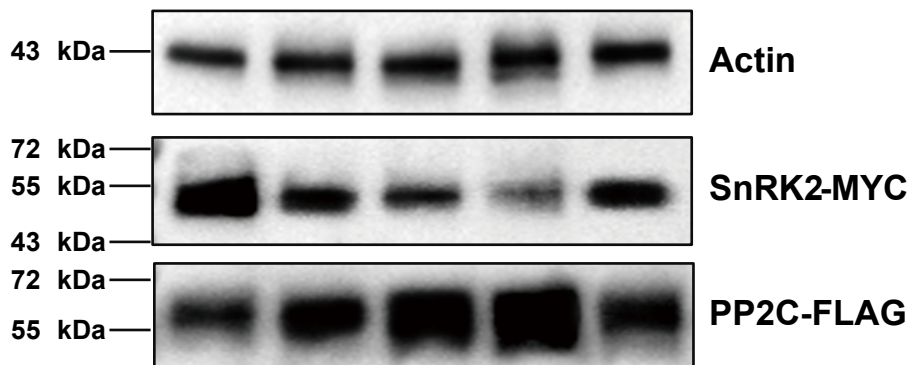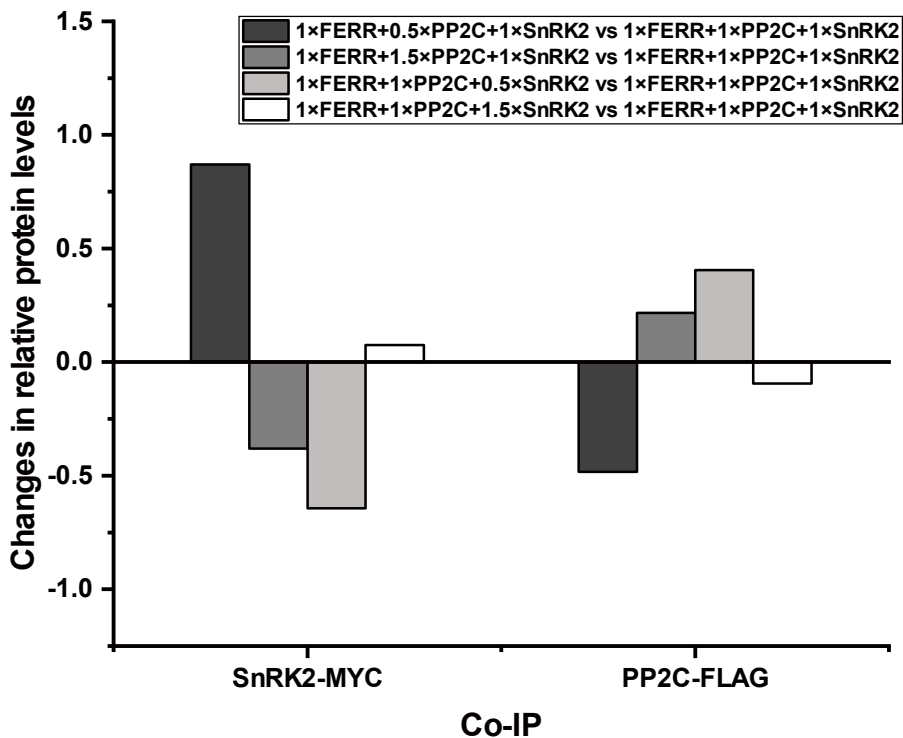

Supplement: Web_Material_uhaf066 [file web_material_uhaf066.zip › Figure S7.pdf]
